# Inhalable hybrid nanovaccines with virus-biomimetic structure boost protective immune responses against SARS-CoV-2 variants

**DOI:** 10.1186/s12951-024-02345-3

**Published:** 2024-02-27

**Authors:** Shuqi Wang, Peiyang Ding, Lingli Shen, Daopeng Fan, Hanghang Cheng, Jian Huo, Xin Wei, Hua He, Gaiping Zhang

**Affiliations:** 1 College of Veterinary Medicine, International Joint Research Center of National Animal Immunology, Henan Agricultural University, Zhengzhou, 450046 China; 2 Longhu Laboratory, Zhengzhou, 450046 China; 3 School of Life Science, Zhengzhou University, Zhengzhou, 450046 China; 4 School of Advanced Agriculture Sciences, Peking University, Beijing, 100871 China; 5 Joint National Laboratory for Antibody Drug Engineering, Henan University, Kaifeng, 475004 China

**Keywords:** SARS-CoV-2, Inhalable hybrid nanovaccine, Genetically engineered nanovesicles, Virus-biomimetic structure, Mucosal immunity, Broad-spectrum neutralization activity

## Abstract

**Background:**

Severe acute respiratory syndrome coronavirus 2 (SARS-CoV-2), with different antigenic variants, has posed a significant threat to public health. It is urgent to develop inhalable vaccines, instead of injectable vaccines, to elicit mucosal immunity against respiratory viral infections.

**Methods:**

We reported an inhalable hybrid nanovaccine (NV_RBD_-MLipo) to boost protective immunity against SARS-CoV-2 infection. Nanovesicles derived from genetically engineered 293T cells expressing RBD (NV_RBD_) were fused with pulmonary surfactant (PS)-biomimetic liposomes containing MPLA (MLipo) to yield NV_RBD_-MLipo, which possessed virus-biomimetic structure, inherited RBD expression and versatile properties.

**Results:**

In contrast to subcutaneous vaccination, NV_RBD_-MLipo, via inhalable vaccination, could efficiently enter the alveolar macrophages (AMs) to elicit AMs activation through MPLA-activated TLR4/NF-κB signaling pathway. Moreover, NV_RBD_-MLipo induced T and B cells activation, and high level of RBD-specific IgG and secretory IgA (sIgA), thus elevating protective mucosal and systemic immune responses, while reducing side effects. NV_RBD_-MLipo also demonstrated broad-spectrum neutralization activity against SARS-CoV-2 (WT, Delta, Omicron) pseudovirus, and protected immunized mice against WT pseudovirus infection.

**Conclusions:**

This inhalable NV_RBD_-MLipo, as an effective and safe nanovaccine, holds huge potential to provoke robust mucosal immunity, and might be a promising vaccine candidate to combat respiratory infectious diseases, including COVID-19 and influenza.

**Supplementary Information:**

The online version contains supplementary material available at 10.1186/s12951-024-02345-3.

## Introduction

Coronavirus disease 2019 (COVID-19), caused by severe acute respiratory syndrome coronavirus 2 (SARS-CoV-2) with different antigenic variants, has spread rapidly around the world, thus threatening public health, financial and social systems [1–3]. Respiratory tract is the primary site of SARS-CoV-2 transmission, where SARS-CoV-2 enters the host cells through viral spike (S) protein binding to angiotensin-converting enzyme 2 (ACE2) receptor [4–6]. Therefore, it is believed that introduction of mucosal immunity as the first-line of defense is essential for effective resistance to SARS-CoV-2 infection. However, multiple approved COVID-19 vaccines, such as inactivated virus vaccines [7], recombinant protein subunit vaccines [8, 9], and messenger (mRNA) vaccines [10], are suboptimal in producing secretory IgA (sIgA) to neutralize viruses in the respiratory tract, leading to limited mucosal immune response [11, 12].

To date, inhalable COVID-19 vaccines have drawn great attention, which can evoke a significant mucosal immune responses in the mucosa-associated lymphoid tissue (MALT), especially nasal-associated lymphoid tissue (NALT) [13, 14], and antibody titers to neutralize virus at the first line, while also showing advantages for needle-free and non-invasive administration [15]. For example, Convidecia Air, as an inhalable COVID-19 vaccine has been approved for use in China [16]. Especially, inhalable recombinant protein vaccine, which contains antigen (e.g., receptor-binding domain (RBD)) from pathogen, has been extensively studied as an attractive vaccine candidate due to their safety [17, 18]. However, antigen production is time-consuming and expensive, premature degradation and low immunogenicity of antigen require incorporation of a suitable adjuvant to reduce the number of vaccinations and boost immune responses [19, 20]. Monophosphoryl lipid A (MPLA) is a well-known toll-like receptor 4 (TLR4) agonist, which plays a critical role in the induction of innate and adaptive immune responses through activation of TLR4/nuclear factor kappa-B (NF-κB) signaling pathway [21–24]. In addition, as one of the most common, safe and approved adjuvants, Alum adjuvant can enhance antigen immunogenicity, whereas the protective efficacy of Alum-formulated vaccine is limited due to the lack of cellular immunity [25, 26]. Although other adjuvants have demonstrated promising efficacy, they have not been approved for widespread use due to safety concerns [27]. Alternatively, pulmonary surfactant (PS) that is an aggregate of lipid and surfactant proteins (SP-A, SP-B, SP-C and SP-D), acts as a natural biological barrier for the respiratory surface, thus imposing restrictions on the entry of foreign nanoparticles and hydrophilic molecules into the alveolar macrophages (AMs) [28–30]. As such, it is highly imperative to develop inhalable vaccine delivery systems with potent antigen presentation, adjuvant and PS-penetrating capabilities to boost mucosal immunity.

Cell-derived nanovesicles (NVs), which are prepared by sonicating or extruding cell membrane, have gained considerable attention as a promising candidate to extend the potential of vaccine delivery systems [31–35]. NVs incorporate the lipids and proteins of original parent cells, which assist in inheriting unique biological properties of source cells [36–38]. It has been proved in multiple studies that NVs possess significant advantages over conventional delivery carriers, including excellent biocompatibility and prolonged circulation time [39]. However, poor cargo-loading efficacy and low stability have hampered the translational progress of NVs [40, 41]. To address these challenges, genetic engineering of cell has been shown to be an effective approach, which allows for selective expression of specified protein, especially antigen, thus further optimizing the antigen delivery efficiency of NVs [42]. In addition, liposome has been served as promising candidate for vaccine carriers thanks to their excellent merits of high stability, cargo-loading capacity and versatile structure modification [43–46]. Encouragingly, PS-biomimetic liposomes that composed of 1,2-dipalmitoyl-*sn*-glycero-3-phosphocholine (DPPC)/dipalmitoyl phosphatidylglycerole (DPPG)/1,2-dipalmitoyl-*sn*-glycero-3-phosphoethanolamine (DPPE)-COOH/cholesterol can enter AMs *via* pulmonary surfactant-associated protein (SP)-A/D-mediated endocytosis with negligible PS layer disruption [47]. Thus, we hypothesized that hybrid NVs-liposomes nanoplatforms can be fabricated based on their similar structures to accomplish complementary and versatile performance. In this regard, the hybrid nanoplatforms can inherit both the features of biological properties and antigen expression from genetically engineered NVs, as well as PS-penetrating ability of liposomes.

**Scheme 1:**
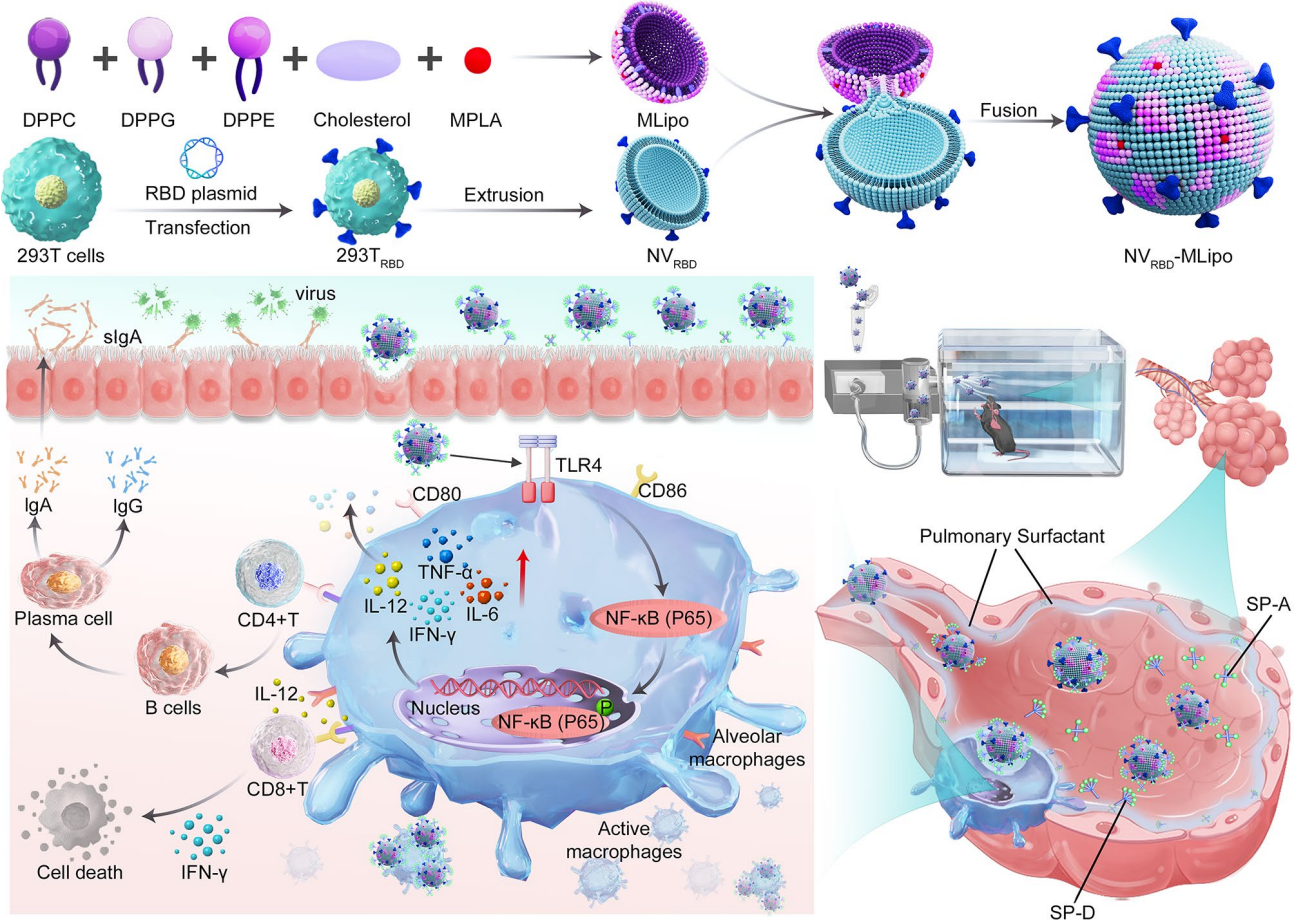
Schematic illustration of inhalable hybrid nanovaccines (NV_RBD_-MLipo) with virus-biomimetic structure against SARS-CoV-2 infection. Nanovesicles are derived from genetically engineered 293T cell expressing RBD (NV_RBD_), which are further fused with pulmonary surfactant (PS)-biomimetic liposomes containing MPLA (MLipo) to yield NV_RBD_-MLipo. After inhalable vaccination, NV_RBD_-MLipo effectively enters alveolar macrophages (AMs) due to its PS-biomimetic property, and induces AMs activation through MPLA-activated TLR4/NF-κB signaling pathway. Furthermore, NV_RBD_-MLipo elicits T and B cells activation, as well as generates high level of RBD-specific IgG and secreted IgA (sIgA) with broad-spectrum neutralization activity against pseudoviruses, thus boosting protective mucosal immune and systemic immune responses

Herein, we developed inhalable hybrid nanovaccines (known as NV_RBD_-MLipo) with virus-biomimetic structure to provide effective protection against SARS-CoV-2 variants (Scheme 1). The NV_RBD_-MLipo was fabricated by efficient fusion of genetically engineered NVs expressing RBD (known as NV_RBD_) and MPLA-containing, PS-biomimetic liposomes (known as MLipo) with long-term PBS or serum stability. The obtained NV_RBD_-MLipo maintained the similar RBD expression to NV_RBD_, which mimicked the surface distribution of specific antigen on virus, thus enabling the effective antigen presentation. Compared with reported subcutaneous COVID-19 vaccines, inhalable NV_RBD_-MLipo which inherited the capabilities of liposomes and source cells, could efficiently enter the AMs and elicit AMs activation through MPLA -activated TLR4/NF-κB signaling pathway, generate high titer of RBD-specific IgG in serum and RBD-specific secretory IgA (sIgA) in bronchoalveolar lavage fluid (BALF), induce lung and spleen T cells activation, thus potentiating mucosal immunity and systemic (humoral, cellular) immunity, while minimizing side effects. During mucosal immune excitation, sIgA generated by effector B cells could effectively neutralize viruses in the respiratory tract, thus suppressing viral adhesion to and invasion of recipient cells. Therefore, inhalable NV_RBD_-MLipo nanovaccines might provide additional perspective in the development of effective and safe vaccine against respiratory infectious diseases, including SARS-CoV-2 and influenza.

## Methods

### Preparation of NV_RBD_

The mixture of RBD plasmid (2.5 µg) and Lipo6000™ (5 µL) was dissolved in DMEM (250 µL), and incubated for 5 min at room temperature (RT) according to the manufacturer’s protocol [48]. 293T cells seeded on 6-well plates (~ 80% confluence) were incubated with the mixture (2.5 µg RBD plasmid/well) for 24 h before collecting by centrifugation (1000 rpm, 5 min, 4 ℃). The collected cells were resuspended in 1 mM PBS, and freeze-thawed for three times. After centrifugation (14,800 rpm, 30 min, 4 ℃), the resulting precipitate was resuspended in DI water and extruded by Avanti Polar Lipids to obtain RBD-expressed 293T cell-derived nanovesicles (NV_RBD_). The RBD content was determined by enzyme-linked immunosorbent assay (ELISA) kit.

### Preparation of NV_RBD_-MLipo

MPLA-modified liposomes (MLipo) were prepared using the thin-film hydration methods [49]. Briefly, DPPC (13 mg), DPPE (2 mg), DPPG (2 mg), cholesterol (1 mg) and MPLA (0.4 mg) were dissolved in chloroform (2 mL), and the mixture was evaporated by a rotary evaporation to yield a thin film. The dried film was hydrated with PBS, stirred at 40 ℃ for 30 min, and extruded by Avanti Polar Lipids to obtain MLipo. Subsequently, NV_RBD_ was mixed with MLipo at the mass ratio of 1:1. After sonication for 4 min, the mixture was stirred at 37 ℃ for 30 min, and further extruded as described above to yield hybrid liposomes fused with NV_RBD_ (NV_RBD_-MLipo). Cationic liposomes (NV_RBD_-MLipo (+)) that DPPG was replaced with DOTAP were prepared following the above method.

### Characterization of NV_RBD_-MLipo

The particle size, zeta potential and morphology of NV_RBD_, MLipo and NV_RBD_-MLipo were measured by dynamic light scattering instrument (DLS) and transmission electron microscopy (TEM), respectively. To assess the stability, NV_RBD_, MLipo and NV_RBD_-MLipo were co-incubated with DMEM containing 10% FBS or PBS at RT for various time before the measurement of particle size.

In order to evaluate the fusion between liposomes and nanovesicles, MLipo and NV_RBD_ were labeled with rhodamine B (RB) and fluorescein isothiocyanate (FITC), respectively. After fusion, the RB/FITC-NV_RBD_-MLipo was subjected to flow cytometric analysis and confocal laser scanning microscope (CLSM) observation. The physical mixture of RB-MLipo and FITC-NV_RBD_ served as a control. Alternatively, the Fourier-transform infrared (FTIR) spectroscopy was performed on the instrument (PerkinElmer) to characterize the structure of NV_RBD_, MLipo, and NV_RBD_-MLipo. To determine RBD expression on the NV_RBD_, MLipo and NV_RBD_-MLipo, samples were lysed in RIPA buffer, centrifuged (12,000 rpm, 20 min) to collect protein. The RBD expression was detected via Western blot and ELISA as described previously [50]. Na/K-ATPase was used as an internal control.

### Macrophage uptake in vitro

To evaluate the macrophage uptake under PS treatment, RB-NV_RBD_-MLipo or RB-NV_RBD_-MLipo (+) (0.05 mg/mL) was mixed with PS for 1 h. RAW 264.7 cells seeded on 24-well plates (~ 80% confluence) were incubated with the mixture for 4 h, stained with DAPI (5 µg/mL) and observed by CLSM. Alternatively, cells were treated with the mixture for 4 h as above and lysed with RIPA buffer. The RB and protein contents were detected by spectrofluorimetry (λ_ex_ = 488 nm, λ_em_ = 525 nm) and BCA kit, respectively. NVs without PS treatment were used as controls. In addition, cells that treated with different groups for 30 min as described above were washed three times with PBS, collected and used for flow cytometric analysis.

### In vitro macrophage activation

RAW 264.7 cells seeded on 24-well plates (~ 80% confluence) were incubated with different groups (0.05 mg RBD/mL, 0.25 mg Lipo/mL) for 12 h. The collected cells were stained with anti-CD86-FITC and anti-CD80-FITC for 30 min, and then detected by flow cytometry. The concentration of IL-6, IL-12, TNF-α and IFN-γ in culture supernatants was determined by ELISA kits according to the experimental protocol.

### In vivo biodistribution

C57BL/6 mice were administered Cy5.5-RBD, Cy5.5-NV_RBD_-MLipo (+), Cy5.5-NV_RBD_-MLipo via inhalation (nebulization, N) (25 µg RBD/mouse, *n* = 3). Mice that were subcutaneously immunized with Cy5.5-NV_RBD_-MLipo in the mice back skin served as controls. At different time points, fluorescence imaging was obtained using In vivo imaging system (IVIS® Lumina III, PerkinElmer, USA). In addition, the heart, liver, spleen, lung and kidney of mice were collected at 12 h post injection, and subjected for ex vivo imaging.

To further assess the in vivo uptake of NV_RBD_-MLipo in AMs, mice were administered different groups labeled with FITC as described above (25 µg RBD/mouse, *n* = 3), and sacrificed at 12 h post injection. The lungs were collected, frozen in Optimal Cutting Temperature (OCT) embedding compound, sectioned, stained with DAPI for fluorescence microscope imaging. Alternatively, pneumonocyte was stained with anti-CD11b-APC and analyzed by flow cytometry.

### Mice vaccination in vivo

C57BL/6 mice were randomly assigned to five groups, and immunized with PBS, RBD, NV-MLipo, and NV_RBD_-MLipo via inhalation (nebulization, N) on day 0, 7, 14 (25 µg RBD/mouse, *n* = 5). Mice with subcutaneous injection (Sc) of NV_RBD_-MLipo served as controls. The body weight of mice was monitored every two days. On day 18, immunized mice were sacrificed, and serum, BALF, nasal wash, spleen and lung were collected. Moreover, nasal wash, BALF, and serum were collected at day 28, 42 for further use.

To evaluate the AMs activation, the pneumonocytes of immunized mice were collected on day 18. Pneumonocyte were stained with anti-CD11b-APC, anti-CD80-FITC and anti-CD86-FITC for 30 min before flow cytometric analysis. In addition, the concentration of IL-6, TNF-α and IL-12 in BALF was detected using ELISA kit.

### RBD-specific IgG and IgA antibody detection

The antibody titer assay was performed using an indirect ELISA method [51]. Briefly, the 96-well microtiter plates were coated with RBD protein (5 µg/mL), stored at 4 ℃ overnight, and washed three times with PBS. The serial dilutions of collected serum were added into wells, and further incubated at 37 ℃ for 2 h. Then, the plates were washed three times with PBS, and incubated with HRP-conjugated IgG, IgG2c, IgG1 secondary antibody at 37 ℃ for 1 h, respectively. After three washes with PBS, TMB solution was added and incubated for 15 min. Finally, the stop solution (2M H_2_SO_4_) was added and the absorbance at 450 nm was measured by microplate reader. The positive data were defined 2.1 times more than the negative data. The RBD-specific IgA from BALF or nasal wash was determined following a similar protocol as described above. HRP-conjugated IgA was used as secondary antibody.

### Humoral immunity in vivo

To evaluate B cells responses, spleens were collected from immunized mice on day 18, and splenocytes were prepared by passing the spleen through 40 μm cell strainer, followed by red blood cell (RBC) lysis. After three washes with PBS (0.1 M), splenocytes were collected, stained with anti-IgD-FITC and anti-CD19-APC for 30 min, and detected by flow cytometry. Alternatively, pneumonocytes were also isolated on day 18 and stained with anti-IgD-FITC and anti-CD19-APC before flow cytometric analysis.

### Cellular immunity in vivo

To investigate the T cells responses, pneumonocytes or splenocytes were collected from immunized mice on day 18 as described above, stained with anti-CD3-APC and anti-CD4-FITC for 30 min before flow cytometric analysis. To further evaluate the RBD-specific CD8^+^ T cell activation, splenocytes and pneumocytes were co-cultured with RBD (5 µg/well) for 4 h, respectively. After blocking with 0.1% BSA to inhibit extracellular cytokine secretion, cells were stained with anti-CD8-FITC, and fixed with fixation buffer. Subsequently, cells were permeabilized in permeabilizing buffer and stained with anti-IFN-γ-APC before flow cytometric analysis.

To assess the proliferation of splenocytes, splenocytes seeded on 96-well plates (~ 80% confluence) were retreated with RBD (5 µg/well) for 48 h. The cells were counted, and the IFN-γ level in the culture medium was measured via ELISA kit. Alternatively, the splenocytes were seeded on 96-well IFN-γ enzyme-linked immunosorbent spot (ELISPOT) plates at 5 × 10^5^ cells/well, and further re-stimulated with RBD (5 µg/mL). After incubation for 48 h, the cells were measured according to the manufacturer’s protocols.

### Pseudovirus neutralization

C57BL/6 mice were immunized with PBS, and RBD, NV-MLipo, and NV_RBD_-MLipo via inhalation (nebulization, N) or subcutaneous injection (Sc) on days 0, 7, and 14 following the protocol described above (25 µg RBD/mouse, *n* = 3). Two weeks after the final vaccination, mice were challenged intranasally with DiD-labelled SARS-CoV-2 WT pseudovirus (2.5 × 10^4^ TCID_50_, 50 µL). Mice were imaged once a day using the In vivo Imaging System, and lungs collected on day 34 were subjected for ex vivo imaging. In addition, the collected lungs were embedded in OCT, sectioned, stained with DAPI for visualization by fluorescence microscopy.

To evaluate neutralization activity against SARS-CoV-2 WT, Delta, Omicron pseudovirus, sera were collected from immunized mice on day 28, serially diluted with DMEM containing 10% FBS, and incubated with SARS-CoV-2 pseudovirus at 37 ℃ for 1 h. Vero cells seeded on 96-well plates (~ 80% confluence) were incubated with the mixture for 1 h. Afterwards, the medium was replaced by DMEM containing 2% FBS and cells were further incubated for another 48 h. The luciferase signal of the infected cells was measured by Nano-Glo® Luciferase Assay System (Promega).

### In vivo safety

C57BL/6 mice were immunized with PBS and NV_RBD_-MLipo via inhalation (25 µg RBD/mouse, *n* = 3). To evaluate the acute toxic effects, lung, heart, kidney, liver, brain, spleen were collected on day 14 to calculate the organ coefficients (organ weight/body weight). Besides, blood was also collected to detect globulin (GLOB), albumin (ALB), alanine transferase (ALT), blood urea nitrogen (BUN), alkaline phosphatase (ALKP), total protein (TP) levels.

### Mechanism of immune response

RAW 264.7 cells seeded on 24-well plates (~ 80% confluence) were incubated with RBD and NV_RBD_-MLipo for 12 h (0.25 µg RBD/well), stained with anti-TLR4-PE for 30 min before flow cytometric analysis. Alternatively, the RBD or NV_RBD_-MLipo treated cells were lysed in RIPA buffer on ice for 20 min. After collecting by centrifugation (12,000 rpm, 4 ℃, 20 min), the NF-κB protein expression in the supernatants was detected by Western blot.

For transcriptomic analysis, C57BL/6 mice were immunized with RBD and NV_RBD_-MLipo via inhalation (nebulization, N) (25 µg RBD/mouse, *n* = 3). The pneumonocyte was harvested 12 h after immunization, and total RNA was extracted using Trizol and subjected to high-throughput sequencing (Novogene, Beijing, China). Data were performed by Novo Magic.

### Statistical analysis

Statistical analysis was performed using GraphPad Prism 9 (GraphPad). All flow cytometry data were analyzed using FlowJo v10 software. Data could be expressed as mean ± standard deviation. The student’s t-test was used for statistical comparisons. The differences were set to be significant at *p < 0.05 and very significant at **p < 0.01, ***p < 0.001, and ****p < 0.0001.

## Results and discussion

### Preparation and characterization of NV_RBD_-MLipo

**Fig. 1:**
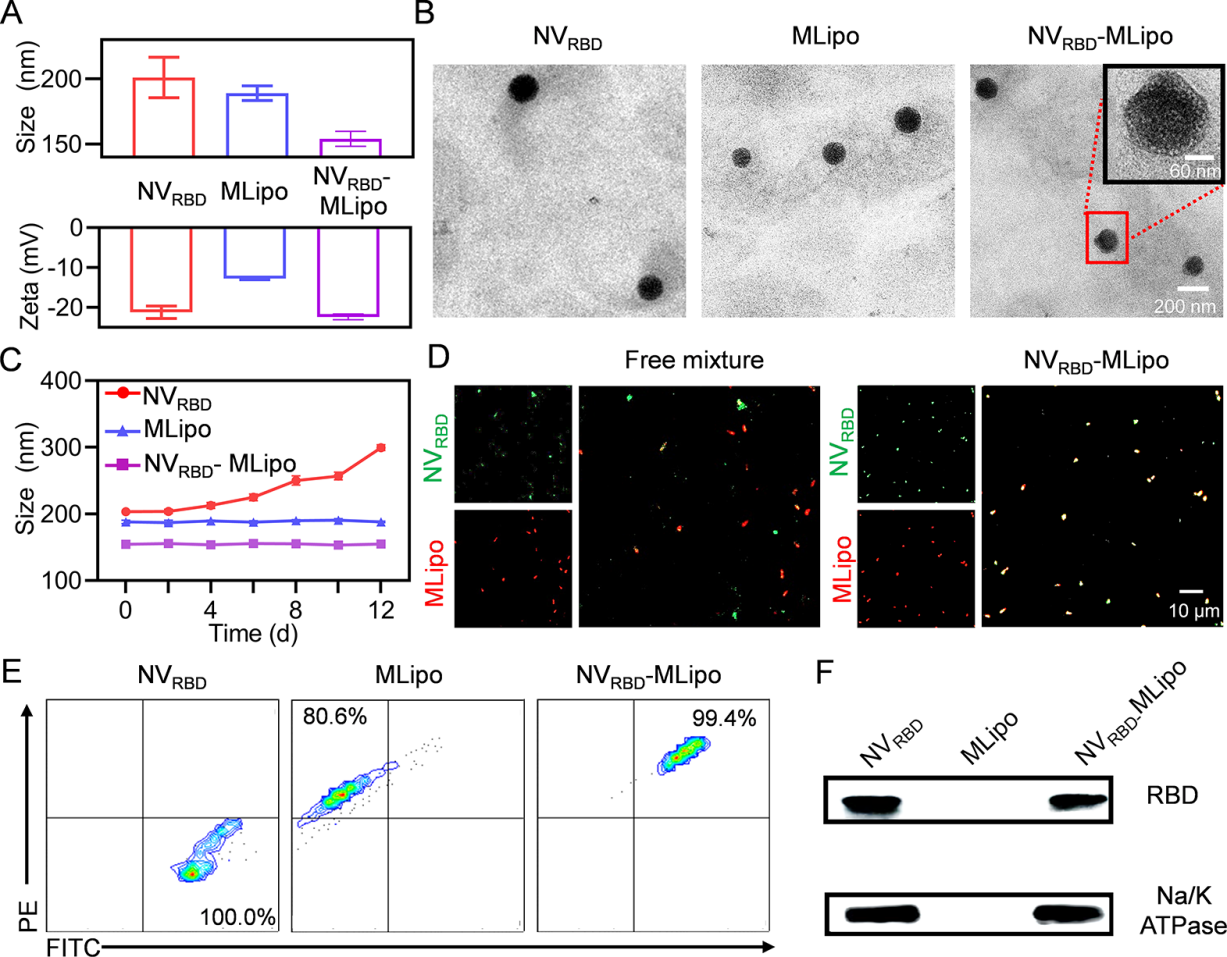
Characterization of hybrid NV_RBD_-MLipo. The size, zeta potential (**A**) and TEM images (**B**) of NV_RBD_, MLipo and NV_RBD_-MLipo (*n* = 3). (**C**) Alternation of particle size of NV_RBD_, MLipo, NV_RBD_-MLipo in serum (*n* = 3). (**D**) CLSM images of physical mixture (NV_RBD_ and MLipo) and NV_RBD_-MLipo. (red, Rhodamine B-labelled MLipo; green, FITC-labelled NV_RBD_). (**E**) FCM analysis of membrane fusion in NV_RBD_-MLipo. (**F**) Western blot analysis of RBD protein expression in NV_RBD_, MLipo and NV_RBD_-MLipo

The preparation of NV_RBD_-MLipo was illustrated in Scheme 1, including the preparation of 293T cells expressing RBD-derived nanovesicles (NV_RBD_) and liposomes, membrane fusion between NV_RBD_ and liposomes. 293T cells were transfected with RBD plasmids (Additional file 1: Fig. S1) for 24 h, purified and extruded to yield the genetic engineering NV_RBD_. As shown in Fig. 1A, the particle size and zeta potential of NV_RBD_ were 201 nm and − 21.2 mV, respectively. Moreover, the RBD band from NV_RBD_ was similar to that of RBD protein (Additional file 1: Fig. S2A). ELISA assay revealed that the content of RBD in the NV_RBD_ was 310 µg/mg NV_RBD_ (Additional file 1: Fig. S2B). These results consistently indicated that RBD was extensively expressed on the NV_RBD_. Alternatively, liposome containing MPLA (MLipo) with the particle size of 189 nm and zeta potential of -12.8 mV was fabricated by thin film dispersion (Fig. 1A), and NV_RBD_-MLipo was further prepared through sonication-triggered membrane fusion of NV_RBD_ with MLipo. Other nanovaccines were prepared following the similar approach, and the abbreviations were listed in Additional file 1: Table S1. At the NV_RBD_/MLipo mass ratio of 1:1, NV_RBD_-MLipo possessed an ideal particle size, zeta potential (154 nm, -22.4 mV) and spherical morphology in TEM images (Fig. 1B and Additional file 1: Table S2), which was close to that of SARS-CoV-2 virus (~ 140 nm). Hence, the optimized NV_RBD_/MLipo mass ratio of 1 was performed for all studies unless otherwise specified. Compared to MLipo, NV_RBD_-MLipo exhibited a smaller particle size and more negative zeta potential, as a result of the introduction of negatively charged NV_RBD_. Moreover, the particle size was maintained when NV_RBD_-MLipo was stored in PBS or FBS for up to 12 days, whereas the particle size of NV_RBD_ enlarged from 201 to 302 nm, indicating that the membrane fusion of NV_RBD_ and liposomes could improve NV_RBD_ stability (Fig. 1C and Additional file 1: Fig. S3).

To evaluate the fusion efficiency of NV_RBD_ and MLipo, NV_RBD_ and MLipo were respectively labeled with FITC and RB, and visualized by CLSM. As shown in Fig. 1D, a divided distribution was observed in the mixture of FITC-NV_RBD_ and RB-MLipo, as evidenced by the separation of red and green fluorescence. In contrast, a significant overlap of red and green fluorescence was demonstrated in NV_RBD_-MLipo, indicating the membrane fusion of NV_RBD_ and MLipo. Flow cytometry further revealed that the PE^+^ FITC^+^ percentage in NV_RBD_-MLipo was higher than that in the mixture, which collectively substantiated that NV_RBD_ was fused to MLipo (Fig. 1E).

The hybrid structure of NV_RBD_-MLipo was further characterized by FTIR spectroscopy. As shown in Additional file 1: Fig. S4, both hydrocarbon region of lipid and signature absorption band of NV_RBD_ (i.e., amide) were presented in the spectrum of NV_RBD_-MLipo, suggesting successful preparation of hybrid NV_RBD_-MLipo. The RBD existence was further examined using Western blot. As illustrated in Fig. 1F, NV_RBD_-MLipo displayed the similar RBD expression to NV_RBD_. Besides, the RBD content was determined to be 104 µg per 1 mg NV_RBD_-MLipo using ELISA method (Additional file 1: Fig. S2C). These results collectively indicated that NV_RBD_-MLipo possessed virus-biomimetic structure and inherited high RBD content of NV_RBD_ through membrane fusion.

### PS-mediated macrophage uptake in vitro

**Fig. 2:**
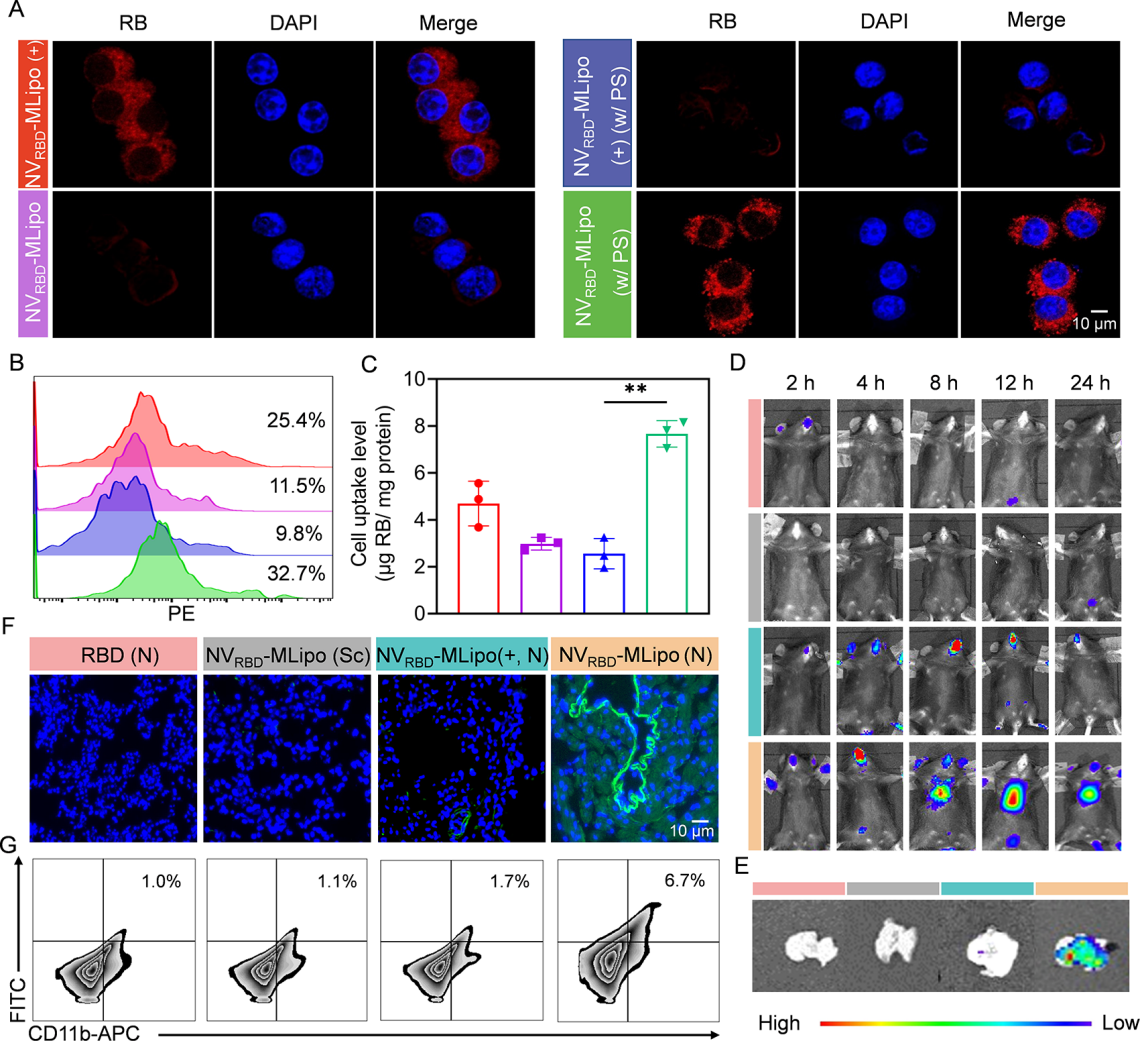
*In vitro* PS-mediated macrophage uptake and in vivo biodistribution. (**A**) CLSM images of RAW 264.7 cells cultured with NV_RBD_-MLipo or NV_RBD_-MLipo (+) for 4 h with or without PS treatment. (**B**) FCM analysis of uptake level of nanovaccines. RAW 264.7 cells were treated with NV_RBD_-MLipo or NV_RBD_-MLipo (+) as described in (**A**). (**C**) The uptake level of nanovaccines (*n* = 3). RAW 264.7 cells were treated with NV_RBD_-MLipo or NV_RBD_-MLipo (+) as described in (**A**). (**D**) The in vivo fluorescence imaging of mice immunized with various groups via inhalation (nebulization, N) or subcutaneous (Sc) injection (*n* = 3). Ex vivo fluorescence (**E**) and immunofluorescence (**F**) imaging of lungs at 12 h post vaccination (*n* = 3). (**G**) FCM analysis of various groups uptake in AMs at 12 h post vaccination (*n* = 3). Data expressed as means ± SD. **p < 0.01

To assess the uptake of NV_RBD_-MLipo in macrophages, the cationic liposomes were prepared as controls, where the positive DOTAP replaced the negative DPPG in the liposomes, the other components of NV_RBD_-MLipo and NV_RBD_-MLipo (+) remained constant. As shown in Fig. 2A, NV_RBD_-MLipo (+) had higher internalization than NV_RBD_-MLipo in RAW 246.7 cells, since NV_RBD_-MLipo (+) could effectively adhere on the negatively charged cell membrane. After incubation with PS for 1 h, the poor uptake of NV_RBD_-MLipo (+) was displayed in RAW 246.7 cells, due to aggregation into the negative PS. In contrast, the uptake of NV_RBD_-MLipo increased significantly, as evidenced by stronger intracellular red fluorescence, which was attributed to similar lipid composition and charge to PS. Consistent with the qualitative results, flow cytometry revealed that the cellular uptake level of NV_RBD_-MLipo was markedly increased under PS condition, resulting in 32.7% internalization following a 4 h incubation (Fig. 2B). Furthermore, the NV_RBD_-MLipo uptake (w/ PS) was 2.6- and 3.0-fold, respectively, higher than that of NV_RBD_-MLipo (w/o PS) and NV_RBD_-MLipo (+, w/ PS) after 4 h incubation (Fig. 2C). These results collectively indicated that NV_RBD_-MLipo could enter the macrophage effectively via PS-mediated endocytosis.

### In vivo biodistribution

To evaluate the biodistribution of RBD and various nanovaccines, mice were immunized with Cy5.5-RBD, Cy5.5-NV_RBD_-MLipo (+), Cy5.5-NV_RBD_-MLipo via inhalation (nebulization, N) or subcutaneous injection (Sc), and subjected to in vivo imaging. As shown in Fig. 2D, significantly increased lung accumulation was demonstrated in mice immunized with Cy5.5-NV_RBD_-MLipo via inhalation rather than subcutaneous injection, suggesting that inhalation could contribute to NV_RBD_-MLipo delivery to the lung. Excitingly, the lung accumulation of inhaled Cy5.5-NV_RBD_-MLipo was maximally enhanced after 12 h and maintained for at least 24 h, whereas Cy5.5-NV_RBD_-MLipo (+) could only be retained in the nasal cavity, indicating the satisfying lung accumulation capacity of NV_RBD_-MLipo. Alternatively, the tissues were isolated from immunized mice at 12 h and subjected to ex vivo imaging, quantitative analysis and immunofluorescence staining. Consistent with the above results, the lung accumulation of inhaled Cy5.5-NV_RBD_-MLipo was 21.1- and 43-fold higher than that of Cy5.5-NV_RBD_-MLipo (Sc) and Cy5.5-NV_RBD_-MLipo (+, N), respectively (Fig. 2E and Additional file 1: Fig. S5A). In addition, extensive green fluorescence was demonstrated in inhalable NV_RBD_-MLipo (Fig. 2F). To further investigate the in vivo NV_RBD_-MLipo uptake by AMs, the harvested lung was performed to flow cytometric analysis. As shown in Fig. 2G and Additional file 1: Fig. S5B, the cellular uptake level was 6.7 times higher than that of the free RBD group, confirming that inhalable NV_RBD_-MLipo affording satisfying lung accumulation capacity could effectively deliver RBD into AMs.

### In vitro macrophage activation

**Fig. 3:**
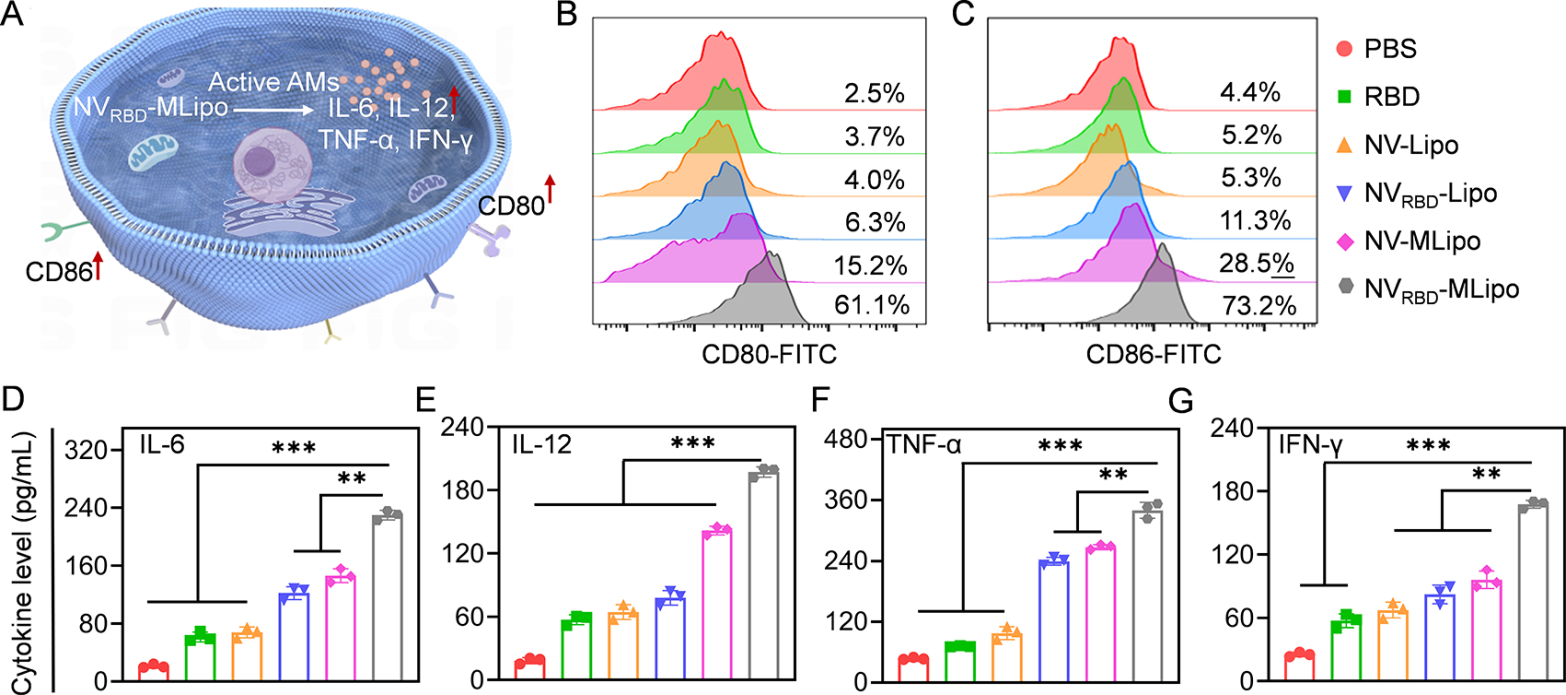
*In vitro* macrophage activation. (**A**) Schematic illustration of macrophage activation. (**B-C**) FCM analysis of activated RAW 264.7 cells following incubation with different groups for 12 h (*n* = 3). The level of IL-6 (**D**), IL-12 (**E**), TNF-α (**F**) and IFN-γ (**G**) secreted by activated RAW 264.7 cells. Cells were treated as described in (B) (*n* = 3). Data expressed as means ± SD. *p < 0.05; **p < 0.01; ***p < 0.001

The nanovaccines-elicited macrophage activation was further evaluated by flow cytometry and ELISA assay (Fig. 3A). RAW 264.7 cells were treated with RBD, NV_RBD_-Lipo, NV-MLipo, NV_RBD_-MLipo under PS condition for 12 h, and analyzed by flow cytometry to determine the level of activated macrophage that overexpressed co-stimulatory molecules, including CD80 and CD86 [52]. Compared with free RBD, NV_RBD_-Lipo (w/o MPLA) or NV-MLipo (w/o RBD) only elicited partial macrophage activation, whereas significantly increased percentage of CD80^+^ and CD86^+^ (16.5- and 14.1-fold, respectively) macrophage was noted in NV_RBD_-MLipo group (Fig. 3B, C), suggesting that NV_RBD_-MLipo was able to elicit significant macrophage activation. Alternatively, the cytokine level in the supernatant harvested from RAW 264.7 cells was further revealed the similar results. IFN-γ can stimulate immune cells activation to interfere virus replication, and IL-6, TNF-α and IL-12 contribute to T cell proliferation and cellular immunity [53, 54]. As shown in Fig. 3D-G, NV_RBD_-MLipo elicited 2.9-4.8-fold higher levels of cytokines (TNF-α, IFN-γ, IL-6, IL-12) than RBD group, respectively. These results collectively identified that NV_RBD_-MLipo could effectively provoke macrophage activation in vitro.

### In vivo AMs activation

**Fig. 4:**
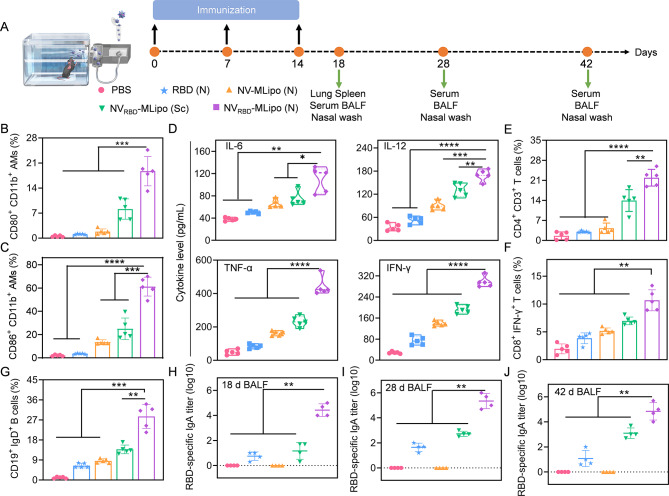
NV_RBD_-MLipo elicited in vivo AMs activation and mucosal immune response. (**A**) Timeline of the in vivo vaccination. Mice were immunized with different groups on days 0, 7, and 14 (25 µg RBD/mouse, *n* = 5). Serum, BALF and nasal wash were collected on days 18, 28 and 42, lung and spleen were collected on day 18. FCM analysis of CD80^+^ CD11b^+^ (**B**), CD86^+^ CD11b^+^ (**C**) AMs proportion in lungs on day 18 (*n* = 5). (**D**)The secretion level of IL-6, IL-12, TNF-α, IFN-γ in the BALF collected on day 18 (*n* = 5). FCM analysis of CD4^+^ CD3^+^ (**E**) and IFN-γ^+^ CD8^+^ (**F**) T cells, CD19^+^ IgD^+^ B cells (**G**) proportion in lungs on day 18 (*n* = 5). RBD-specific IgA titers in BALF assessed by ELISA on days 18 (**H**), 28 (**I**) and 42 (**J**) (*n* = 4). Data expressed as means ± SD. *p < 0.05; **p < 0.01; ***p < 0.001; ****p < 0.0001

In order to evaluate the in vivo AMs activation, randomized mice received three vaccinations with PBS, RBD, NV-MLipo, NV_RBD_-MLipo on days 0, 7, 14 via inhalation (nebulization, N) or subcutaneous injection (Sc), and lungs and BALF were collected from immunized mice on day 18 (Fig. 4A and Additional file 1: Table S3). As shown in Fig. 4B, C and Additional file 1: Fig. S6, immune activation level of AMs was not obvious in RBD or NV-MLipo group due to the lack of nano-adjuvant or RBD (antigen), respectively. Excitingly, higher percentage of CD11b^+^ CD80^+^ and CD11b^+^ CD86^+^ AMs was noted in lungs harvested from NV_RBD_-MLipo (N)-immunized mice, indicating that more AMs were activated by inhalable NV_RBD_-MLipo. To further investigate the effect of different routes of administration on AMs activation, mice were immunized with NV_RBD_-MLipo via Sc injection and the lung was harvested as described above. Due to the fact that the subcutaneously injected NV_RBD_-MLipo would circulate throughout the body rather than respiratory tract and lungs, the activation level of AMs was 2.3–2.5 times low than that via inhalation (Fig. 4B, C). Alternatively, the level of cytokines, including TNF-α, IFN-γ, IL-6, IL-12 in BALF was detected by ELISA. Consistent with flow cytometric results, the maximal cytokine content was shown in NV_RBD_-MLipo (N) group rather than other control groups (Fig. 4D). Collectively, these results indicated that inhalable NV_RBD_-MLipo significantly elicited AMs activation, thus providing a prerequisite for further antibody production and virus neutralization.

### Inhalable NV_RBD_-MLipo elicited mucosal immune response

As the major entry route of SARS-CoV-2 is respiratory tract, strengthening the host’s immune system, especially mucosal immunity through antigen-specific sIgA responses serves as the first line of immunological defense against SARS-CoV-2 infection [54]. After antigens are taken up and presented to T cells by AMs, the activated T cells will further proliferate and differentiate into CD4^+^ T cells that promote the B cells activation and differentiation to produce antibody, and CD8^+^ T cells that can recognize and kill infected cells [55]. To determine the level of T cells activation and differentiation, pneumonocytes were collected from immunized mice on day 18, and subjected to flow cytometric analysis. As demonstrated in Fig. 4E, F and Additional file 1: Fig. S7, significantly increased proportion of CD3^+^ CD4^+^, IFN-γ^+^ CD8^+^ T cells was elicited by NV_RBD_-MLipo (N) rather than controls, suggesting that NV_RBD_-MLipo (N) had potential to assist CD4^+^ and CD8^+^ T cells activation. Moreover, the proportion of CD19^+^ IgD^+^ B cells in NV_RBD_-MLipo (N) was 2.1-27-fold higher than that in the other groups (Fig. 4G, and Additional file 1: Fig. S8), which contributed to produce more antibodies for neutralizing virus.

To evaluate mucosal immune response, nasal wash and BALF of immunized mice were collected on days 18, 28, and 42, and the titer of RBD-specific sIgA was measured by ELISA. As shown in Fig. 4H-J and Additional file 1: Fig. S9, slightly increased titer of sIgA was seen in mice that were vaccinated with RBD (N) and NV_RBD_-MLipo (Sc). Comparatively, inhalation of NV_RBD_-MLipo effectively elicited the highest titers of sIgA in nasal wash and BALF, and the satisfactory amount of sIgA was maintained for up to 42 days. Notably, the titer of sIgA reached a maximum at day 28, which was 7766-fold higher than that in RBD group. In summary, these data highlighted that inhalable NV_RBD_-MLipo could induce robust titer of RBD-specific sIgA for enhancing mucosal immunity.

### Inhalable NV_RBD_-MLipo elicited humoral responses

**Fig. 5:**
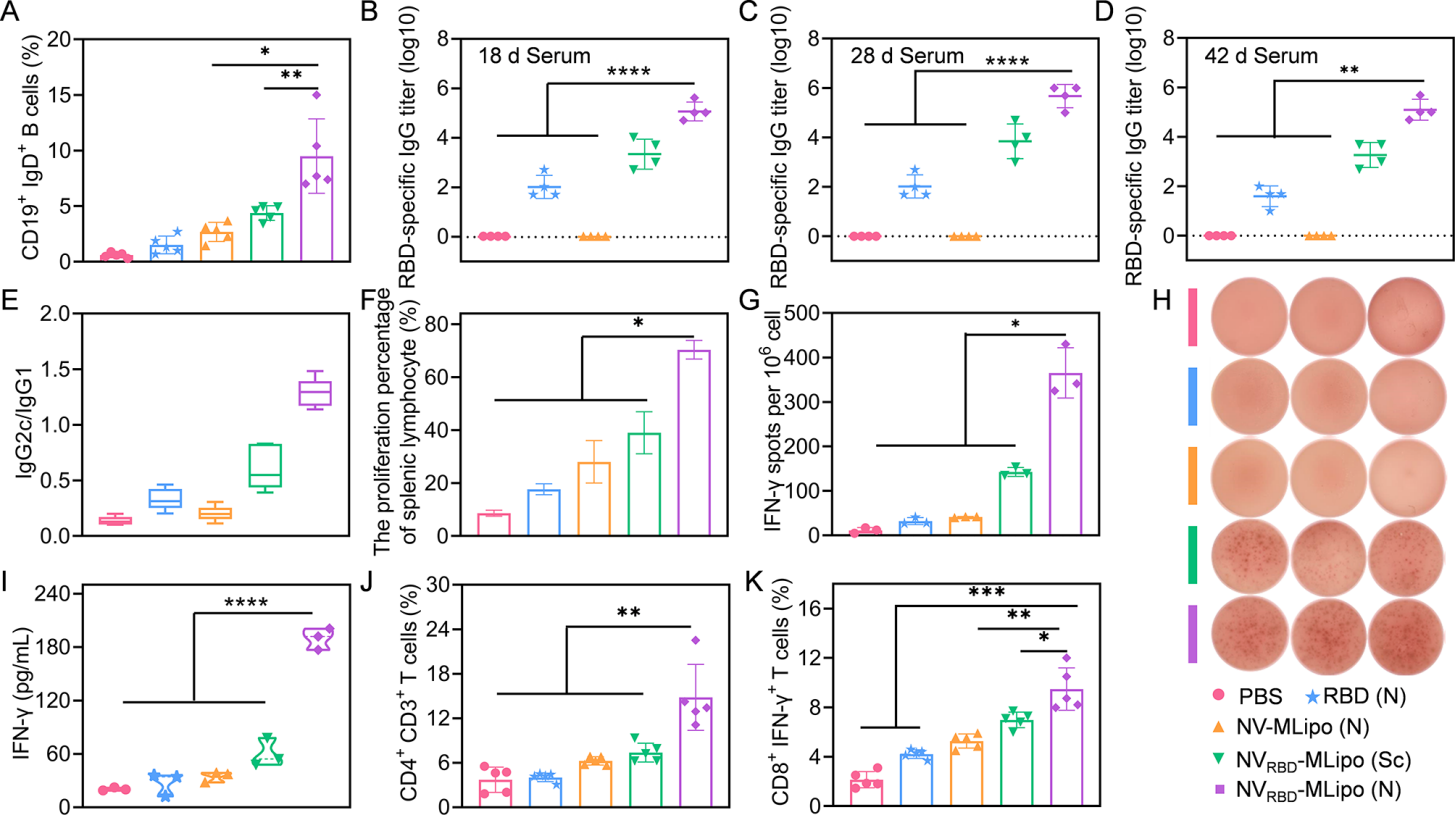
NV_RBD_-MLipo elicited systemic immune response. (**A**) FCM analysis of CD19^+^ IgD^+^ B cells proportion in spleen on day 18 (*n* = 5). RBD-specific IgG titers in serum assessed by ELISA on days 18 (**B**), 28 (**C**) and 42 (**D**) (*n* = 4). (**E**) Ratio of IgG2c to IgG1 in the serum (*n* = 5). (**F**) Proliferation percentage of splenocytes stimulated with RBD on day 18 (*n* = 3). (**G-H**) Representative images and statistical analysis of IFN-γ-producing cells determined by ELISpot (*n* = 3). The cells were collected from the spleen and stimulated with RBD on day 18. (**I**) The secretion level of IFN-γ in the medium of cells stimulated with RBD as described in (**G**). FCM analysis of CD4^+^ CD3^+^ (**J**) and IFN-γ^+^ CD8^+^ (**K**) T cells proportion in spleen on day 18 (*n* = 5). Data expressed as means ± SD. *p < 0.05; **p < 0.01; ***p < 0.001; ****p < 0.0001

B cells are critical for the humoral immunity, which can differentiate into plasma cells to produce antibodies for neutralizing the incoming virus [56]. To evaluate humoral immune response, splenocytes isolated from mice at day 18 were detected by flow cytometry. As shown in Fig. 5A and Additional file 1: Fig. S10, inhalable NV_RBD_-MLipo significantly induced the highest proportion of CD19^+^ IgD^+^ B cells, whereas slightly increased proportion of CD19^+^ IgD^+^ B cells was found in RBD (antigen only), NV-MLipo (adjuvant only), and NV_RBD_-MLipo (Sc) group. Alternatively, the titer of RBD-specific IgG in serum was futher assessed. As shown in Fig. 5B-D, NV_RBD_-MLipo (N) elicited a predominant titer of RBD-specific IgG, which was ~ 1000-fold higher than that of the RBD group at day 28. Excitingly, the satisfactory titer of RBD-specific IgG could be maintained for up to 42 days, which was consistent with sIgA results. In addition, NV_RBD_-MLipo (N) induced Th1-type immune responses, as evidenced by the IgG2c/IgG1 > 1 (Fig. 5E). Collectively, these data indicated that inhalable NV_RBD_-MLipo significantly elicited B cells activation and predominant titer of RBD-specific IgG, thus achieving robust humoral immune responses.

### Inhalable NV_RBD_-MLipo elicited cellular responses

The spleen is one of important peripheral lymphoid organs that plays a critical role in cellular and humoral immunity by providing a site for immune cells to homing and differentiation [57]. To evaluate spleen accumulation, the major organs of mice that received vaccination via inhalation (nebulization, N) or subcutaneous injection (Sc) were harvested at 12 h, and assessed by ex vivo imaging (Additional file 1: Fig. S11). Compared to RBD group with a negligible RBD signal in the spleen, the NV_RBD_-MLipo (N) was also distributed in the spleen in addition to lungs, which was instrumental in boosting immune responses. To further assess the cellular immunity, the spleen of immunized mice was collected on day 18, isolated into single cell suspensions, and re-stimulated with RBD antigen in vitro. Specifically, the proliferation ratio of splenocytes in the NV_RBD_-MLipo (N) was 4-fold higher than that in the RBD group (Fig. 5F). ELISpot assay revealed that IFN-γ-secreting T cells from NV_RBD_-MLipo (N)-immunized mice were dramatically more numerous than those from placebo-immunized mice (Fig. 5G, H). Besides, NV_RBD_-MLipo (N) produced IFN-γ levels that was 6.9-fold higher than those of the RBD group (Fig. 5I). These findings collectively suggested that inhalable NV_RBD_-MLipo could promote T cell proliferation to elicit antigen-specific cellular immunity. Meanwhile, flow cytometry results shown that the significantly increased proportion of CD3^+^ CD4^+^ and IFN-γ^+^ CD8^+^ T cells was found in NV_RBD_-MLipo (N)-immunized mice (Fig. 5J, K and Additional file 1: Fig. S12). Therefore, inhalable NV_RBD_-MLipo robustly induced the cellular immune responses aside from humoral immune responses.

### The neutralizing activity of NV_RBD_-MLipo against SARS-CoV-2 pseudovirus

**Fig. 6:**
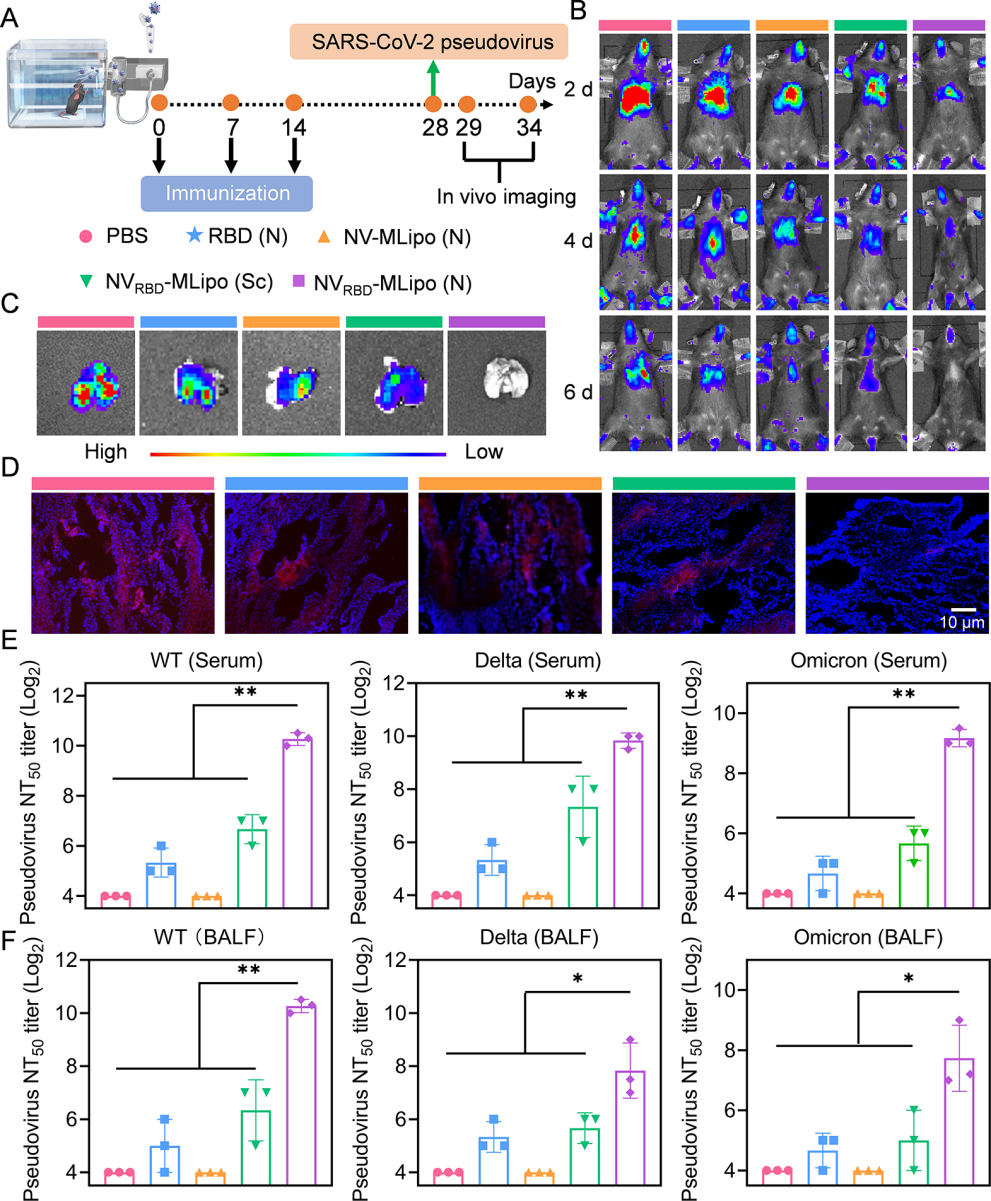
NV_RBD_-MLipo inhibited the infection of SARS-CoV-2 pseudovirus. (**A**) Timeline of in vivo vaccination and challenge. Mice were immunized with different groups via inhalation (nebulization, N) or subcutaneous (Sc) injection on days 0, 7, and 14, and further challenged intranasally with SARS-CoV-2 WT pseudovirus. (**B**) The in vivo fluorescence imaging of immunized mice at different time points post challenge (*n* = 3). Ex vivo fluorescence (**C**) and immunofluorescence (**D**) imaging of lungs at day 6 after challenge (*n* = 3). Serum (**E**) and BALF (**F**) neutralizing capacity against SARS-CoV-2 WT, Delta, Omicron pseudovirus (*n* = 3). The serum and BALF were collected from immunized mice on day 28. Data expressed as means ± SD. *p < 0.05; **p < 0.01

To evaluate whether nanovaccines could neutralize pseudovirus, mice received three vaccination of various groups as described above, and challenged with the DiD-labelled SARS-CoV-2 WT pseudovirus (2.5 × 10^4^ TCID_50_, 50 µL) on day 28 (Fig. 6A). Mice were imaged at different points post challenge to visualize pseudovirus clearance. As demonstrated in Fig. 6B, NV_RBD_-MLipo (N) had potential to promote the rapid clearance of pseudovirus, as evidenced by negligible signal distributed in the lungs. Moreover, the lungs were further harvested on day 34 followed by ex vivo imaging and immunofluorescence staining. Consistent with the above fluorescence imaging results, minimal pseudovirus was found in the lung of mice immunized with NV_RBD_-MLipo (N) compared to other control groups (Fig. 6C, D). Together, these data indicated that inhalable NV_RBD_-MLipo contributed to protect immunized mice from WT pseudovirus infection.

In addition, the pseudovirus neutralization assay was adopted to assess neutralization activity of serum and BALF collected from immunized mice on day 28. As shown in Fig. 6E, F, the serum and BALF from NV_RBD_-MLipo group effectively blocked infection of SARS-CoV-2 WT pseudovirus to Vero cells. Excitingly, the NT_50_ titer (50% neutralization titers) of serum in NV_RBD_-MLipo group was 29.1-fold higher than that in RBD group. The superiority and broad-spectrum of NV_RBD_-MLipo in neutralizing virus were also demonstrated in another two SARS-CoV-2 pseudovirus, such as Omicron and Delta, where NV_RBD_-MLipo again showed 22.8-fold higher NT_50_ titer than RBD. Collectively, these results substantiated that NV_RBD_-MLipo could elicit a high potent, broad neutralization effect against the pseudovirus of SARS-CoV-2 WT, as well as Delta and Omicron variant.

### Safety

**Fig. 7:**
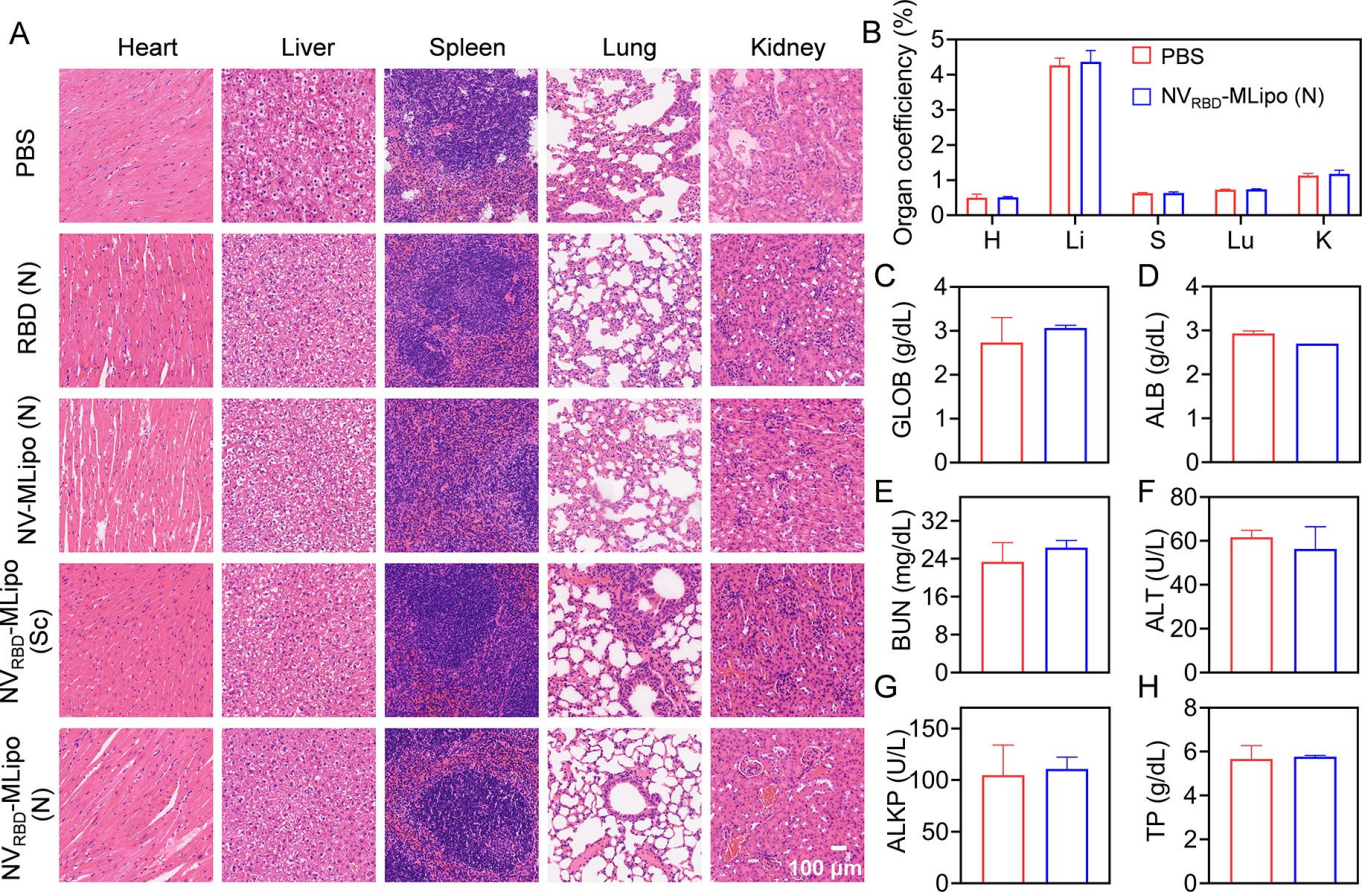
*In vivo* safety. (**A**) H&E staining of major organs sections collected from immunized mice treated as described in Fig. 4 on day 42. Organ coefficient (**B**) and hematological measurement (**C-H**) of mice on day 18 after immunization. (H: Heart; Li: liver; S: Spleen; Lu: Lung; K: Kidney) (*n* = 3)

To evaluate cytotoxicity of NV_RBD_-MLipo, RAW 264.7 cells were incubated with NV_RBD_-MLipo at different concentrations for 12 h. Over 90% cell viability was still remained when the concentration was up to 100 µg/mL, indicating good biocompatibility of NV_RBD_-MLipo (Additional file 1: Fig. S13). In addition, body weight of immunized mice gradually increased (Additional file 1: Fig. S14), and hematoxylin and eosin (H&E) staining demonstrated that there were no pathological abnormalities of major organs (Fig. 7A). 7 days after immunization with NV_RBD_-MLipo, there is no obvious change in the level of GLOB, ALB, BUN, ALT, ALKP, TP (Fig. 7B-H), suggesting that NV_RBD_-MLipo has excellent in vivo safety.

### Mechanism of NV_RBD_-MLipo in regulating immune responses

**Fig. 8:**
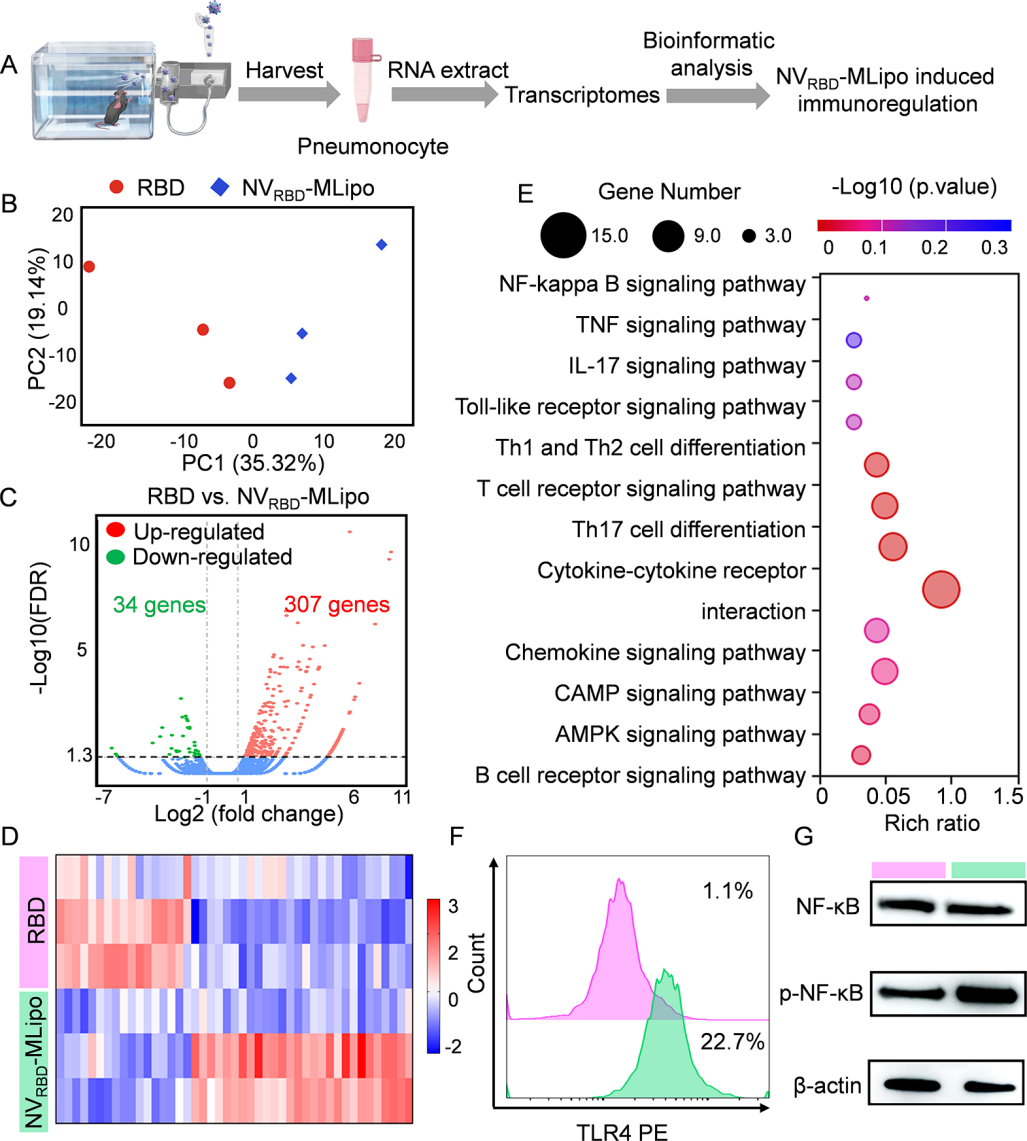
Mechanism of NV_RBD_-MLipo in eliciting immune responses. Lung was harvested from mice at 24 h post immunization and RNA was further isolated for transcriptomic analysis. (**A**) Schematic for transcriptomic analysis of lung. (**B**) Principal component analysis (PCA) of lungs treated with different groups (*n* = 3). Volcano plots (**C**) and heat map (**D**) for differentially expressed genes (DEGs) (*n* = 3). (**E**) KEGG enrichment analysis of DEGs (*n* = 3). (**F**) FCM analysis of TLR4 activation in RAW 264.7 cells following treatment with RBD and NV_RBD_-MLipo for 12 h. (**G**) Western blot analysis of NF-κB and p-NF-κB expression in RAW 264.7 cells. Cells were treated with different groups as described in (**F**)

The molecular mechanism of NV_RBD_-MLipo in activating immune responses in vivo was investigated by the transcriptomes analysis. Mice were immunized with RBD and NV_RBD_-MLipo via inhalation, and the lung was collected at 24 h for RNA sequencing (Fig. 8A). Principal components analysis (PCA) indicated significant differences in total gene expression between RBD and NV_RBD_-MLipo groups (Fig. 8B). Volcano plot and heat map demonstrated that total 341 differentially expressed genes (DEGs) were identified according to the standard of gene expression (fold change > 2, p-adjusted < 0.05), including 307 up-regulated genes and 34 down-regulated genes. Notably, numerous immunoregulatory cytokines (IL-17a) and pro-inflammatory chemokines (CCL12, CCL27a, CXCL9, CXCL10, CXCL12, CXCL13, CXCR5) were found in the up-regulated genes (Fig. 8C, D and Additional file 1: Fig. S15). Moreover, NV_RBD_-MLipo regulated signaling pathways were analyzed by kyoto encyclopedia of genes and genomes (KEGG) enrichment analysis. As shown in Fig. 8E, the up-regulated genes displayed significant enrichment for pro-inflammatory signaling (chemokine signaling pathways, NF-κB pathway, cytokine-cytokine receptor interaction) and T-cell activation signaling (Th1 and Th2 cell differentiation, T-cell receptor signaling pathways, Th17 cell differentiation).

To verify whether NV_RBD_-MLipo could activate the TLR4/NF-κB pathway, RAW 264.7 cells were co-incubated with RBD and NV_RBD_-MLipo for 12 h before flow cytometric and western blot analysis. As shown in Fig. 8F, G, NV_RBD_-MLipo could elicit the activation of TLR4/NF-κB pathway, which was consistent with above transcriptomes analysis results. Collectively, these results suggested that NV_RBD_-MLipo robustly elicit immune responses via TLR4/NF-κB pathway.

## Conclusion

In summary, we developed an inhalable hybrid nanovaccine (NV_RBD_-MLipo) *via* fusing genetically engineered NVs expressing RBD (NV_RBD_) with PS-biomimetic liposomes (MLipo) containing MPLA, which might serve as a safe and effective vaccine candidate for boosting protective immunity against SARS-CoV-2 variants. NV_RBD_-MLipo possessed a virus-biomimetic structure based on inherited advantages of both NV_RBD_ and MLipo, including RBD expression, PS-biomimetic property, and good stability in serum or PBS. Instead of traditional subcutaneous vaccination, inhalable NV_RBD_-MLipo demonstrated a superior ability to elevate internalization into AMs, thus eliciting AMs activation both in vitro and in vivo through MPLA-activated TLR4/NF-κB signaling pathway. Additionally, inhalable NV_RBD_-MLipo displayed superiority over other formulations in increasing the proportion of CD3^+^ CD4^+^ and IFN-γ^+^ CD8^+^ T cells, CD19^+^ IgD^+^ B cells in lung and spleen, producing high titer of RBD-specific IgG in serum and RBD-specific sIgA in BALF and nasal wash, reducing side effects, thus eliciting potent mucosal immunity and systemic (humoral, cellular) immunity, as well as inducing effective, broad-spectrum neutralization activity against SARS-CoV-2 WT, Delta, Omicron pseudovirus, and protecting immunized mice from WT pseudovirus infection. Therefore, this study provides a safe and effective means to prevent and combat respiratory infectious diseases, such as COVID-19 and influenza.

### Electronic supplementary material

Below is the link to the electronic supplementary material.

Supplementary Material 1

## Data Availability

The data used and/or analyzed to support the current study are available from the corresponding author on reasonable request.
