# Supplementary material for: Inhalable hybrid nanovaccines with virus-biomimetic structure boost protective immune responses against SARS-CoV-2 variants
Source: J Nanobiotechnology. 2024 Feb 27;22:76. doi: 10.1186/s12951-024-02345-3 (PMC10898168; doi:10.1186/s12951-024-02345-3)
Supplement: Supplementary file 1 — Supplementary Material 1 [file 12951_2024_2345_MOESM1_ESM.docx]

Supporting Information

Inhalable hybrid nanovaccines with virus-biomimetic structure boost protective immune responses against SARS-CoV-2 variants

Shuqi Wang^1, #^, Peiyang Ding^3, #^, Lingli Shen^1^, Daopeng Fan^1^, Hanghang Cheng^1^, Jian Huo^1^, Xin Wei^5^, Hua He^1,^ *, Gaiping Zhang^1, 2, 4,^ *

^1^College of Veterinary Medicine, International Joint Research Center of National Animal Immunology, Henan Agricultural University, Zhengzhou 450046, China; ^2^Longhu Laboratory, Zhengzhou 450046, China;

^3^School of Life Science, Zhengzhou University, Zhengzhou 450046, China;

^4^School of Advanced Agriculture Sciences, Peking University, Beijing 100871, China;

^5^Henan University Joint National Laboratory for Antibody Drug Engineering, Henan University, Kaifeng 475004, China.

^#^ These authors contributed equally to this work.

* Corresponding author.

E-mail addresses: [hhe@henau.edu.cn](mailto:hehua1123@126.com) (H. He), [zhanggaip@126.com](mailto:zhanggaip@126.com) (G. Zhang).

**Methods**

**Materials, cells, pseudovirus and animals**

Dipalmitoyl phosphatidylglycerole (DPPG), 1,2-dipalmitoyl-*sn*-glycero-3-phosphocholine (DPPC), 1,2-dioleoyl-3-trimethylammoium-propane (DOTAP), cholesterol and 1,2-dipalmitoyl-*sn*-glycero-3-phosphoethanolamine (DPPE) were purchased from Yuanye Biotechnology Co. Ltd (Shanghai, China). Monophosphoryl Lipid A (MPLA) was purchased from Sigma-Aldrich (St Louis, MO, USA). BCA kit, MTT, Lipo6000™ transfection reagent, HRP-labeled Goat Anti-Rabbit IgG (H+L) and anti-Na^+^/K^+^ ATPase α1 were obtained from Beyotime Biotechnology (Shanghai, China). Rhodamine B, FITC, and Cy5.5-NHS were purchased from Aladdin (Shanghai, China). Anti-TNF-α, anti-IL-12, anti-IL-6 and anti-IFN-γ ELISA kits were purchased from Thermo Fisher Scientific (Waltham Mass, USA). Anti-SARS-CoV-2 Spike RBD, anti-CD80-FITC, anti-CD86-FITC, anti-CD11b-APC, anti-CD3-APC, anti-CD4-FITC, anti-CD8-FITC were purchased from Abcam (Cambridge Science Park, UK). Anti-TLR4 (CD284)/MD2-PE, anti-NF-κB, anti-p-NF-κB, anti-CD19-APC, anti-IgD-FITC and anti-IFN-γ-APC were purchased from Biolegend (California, USA). IFN-γ ELISpot kit was purchased from BD Biosciences (New Jersey, USA). HRP-conjugated Affinipure Goat Anti-Mouse IgA (α), IgG1 and IgG2c were purchased from Proteintech (Wuhan, China). All solvents were purchased from Sinopharm Chemical Reagent Co. Ltd (Shanghai, China).

RAW 264.7, Vero and 293T cells were obtained from ATCC (Rockville, MD) and cultured in Dulbecco’s modified Eagle’s medium (DMEM) medium containing 10% fetal bovine serum (FBS). RBD protein was provided by Zhengzhou University. SARS-CoV-2 pseudovirus (WT, Delta, Omicron) was provided by Henan University.

Female C57BL/6 mice (6-8 weeks) were purchased from Animal experimental center of Huaxing (Zhengzhou, China) and housed in clean room. All animal studies were approved by the Institutional Animal Care and Use Committee, Henan Agricultural University.

**Construction of RBD plasmids**

The RBD (319–541 aa) gene was fused to the transmembrane and intracellular regions of the mouse FC receptor using GGGGS as a linker. The fragment was cloned into the pcDNA3.1 vector.

**Synthesis of fluorescence-labeled RBD**

To observe and quantify RBD *in vivo*, the DMSO solution of Cy5.5-NHS (1 mg/mL, 1 mL) was added to the RBD (2 mg/mL, 5 mL) dissolved in PBS (0.1M, pH = 8.3). The mixture was stirred in the dark at room temperature (RT) for 12 h, and then dialyzed against DI water for 2 days (MWCO = 3500 Da). Cy5.5-RBD was obtained after lypophilization.

**Extraction of PS and BALF**

Pulmonary surfactant (PS) were isolated as described previously[1]. Lungs were surgically removed after mice were euthanized, and flushed three times with PBS (1 mL). After centrifugation (400 g, 5 min, 4 ℃), the supernatant and precipitate were collected to obtain bronchoalveolar lavage fluid (BALF) and pneumonocyte, respectively. PS was collected by centrifuging BALF (14800 rpm, 20 min, 4 ℃).

**Cytotoxicity**

RAW 264.7 cells were seeded on 96-well plates at 1 × 10^4^ cells/well and incubated for 12 h. NV_RBD_-MLipo was added at different concentrations and cells were further incubated for 24 h before viability assessment by the MTT assay.

**Reference**

1. Wang J, Li P, Yu Y, Fu Y, Jiang H, Lu M, Sun Z, Jiang S, Lu L, Wu MX. Pulmonary surfactant–biomimetic nanoparticles potentiate heterosubtypic influenza immunity. Science. 2020; 367:869.

**Table S1.** The full name and abbreviations of nanovaccine.

| full name | abbreviation |
| --- | --- |
| nanovesicle derived from 293T cell expressing RBD | NV_RBD_ |
| liposome containing MPLA | MLipo |
| hybrid nanovaccine fused with NV_RBD_ and MLipo | NV_RBD_-MLipo |
| hybrid nanovaccine fused with NV_RBD_ and cationic MLipo | NV_RBD_-MLipo (+) |
| hybrid nanovaccine fused with NV_RBD_ and liposome | NV_RBD_-Lipo |
| hybrid nanovaccine fused with nanovesicle and MLipo | NV-MLipo |

**Table S2.** Particle size and RBD content of NV_RBD_-MLipo at various NV_RBD_/MLipo mass ratios.

| w/w | Size (nm) | Zeta (mV) | RBD content  (μg RBD/mg NV_RBD_-MLipo) |
| --- | --- | --- | --- |
| 0.5 | 158.5 | -21.4 | 41 |
| 1 | 154.9 | -22.4 | 103 |
| 2 | 183.2 | -23.1 | 93 |
| 4 | 212.4 | -23.8 | 82 |

**Table S3.** The dosage and administration route of various groups.

| Group | Antigen/Adjuvant dosage (per mouse) | Administration |
| --- | --- | --- |
| PBS | - | Inhalation |
| RBD | 25 μg RBD | Inhalation |
| NV-MLipo | 5.2 μg MPLA | Inhalation |
| NV_RBD_-MLipo | 25 μg RBD + 5.2 μg MPLA | Subcutaneous injection |
| NV_RBD_-MLipo | 25 μg RBD + 5.2 μg MPLA | Inhalation |


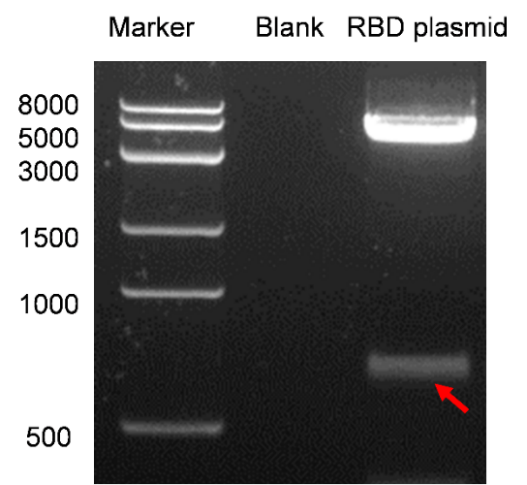


**Fig. S1.** Electrophoresis of RBD plasmid with Hind III and BamH I enzyme digestion.


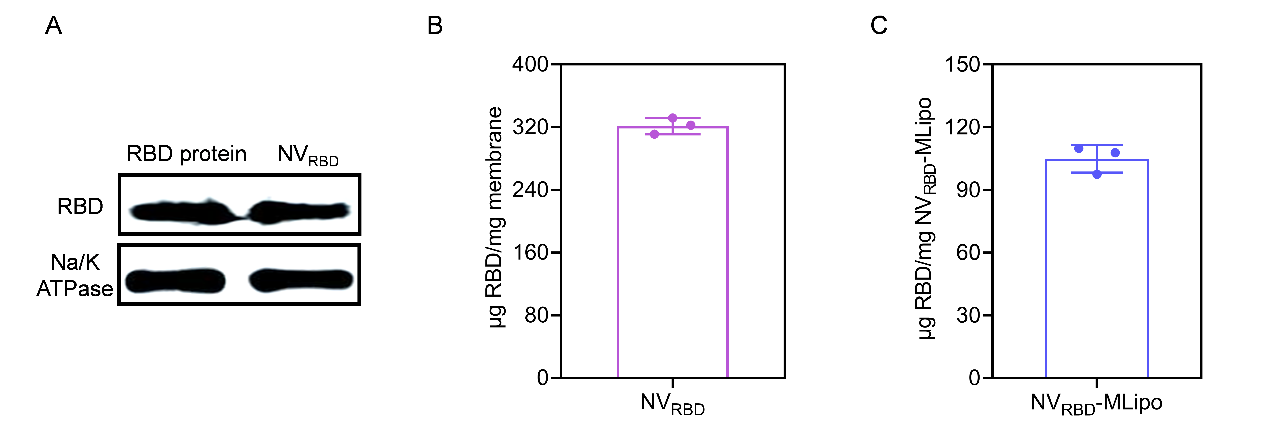


**Fig. S2.** (A) Western blot analysis of RBD protein expression in RBD protein and NV_RBD_. RBD content in NV_RBD_ (B) and NV_RBD_-MLipo (C) determined by ELISA (n = 3).


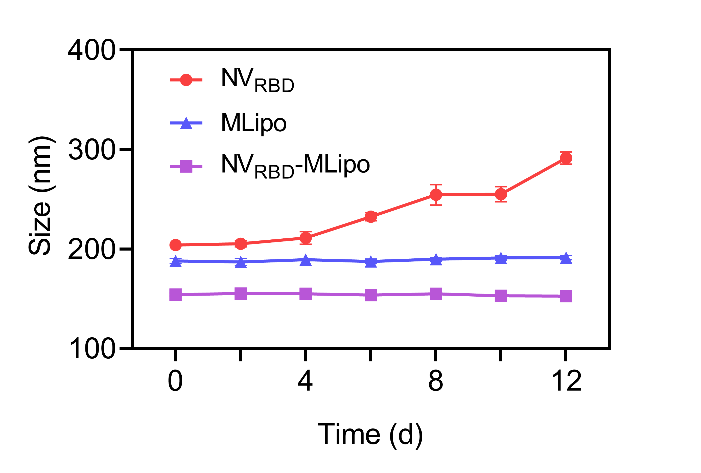


**Fig. S3.** Alternation of particle size of NV_RBD_, MLipo, NV_RBD_-MLipo in PBS (n = 3).


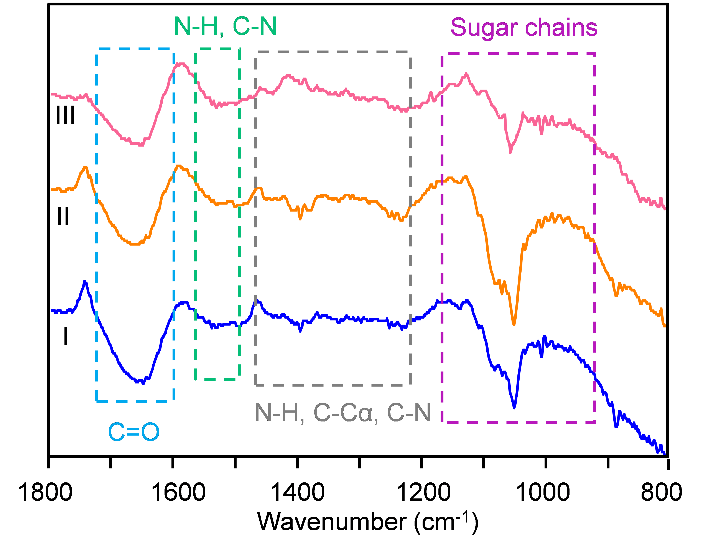


**Fig. S4.** FTIR analysis of membrane fusion in NV_RBD_-MLipo. (I: NV_RBD_, II: MLipo, III: NV_RBD_-MLipo)


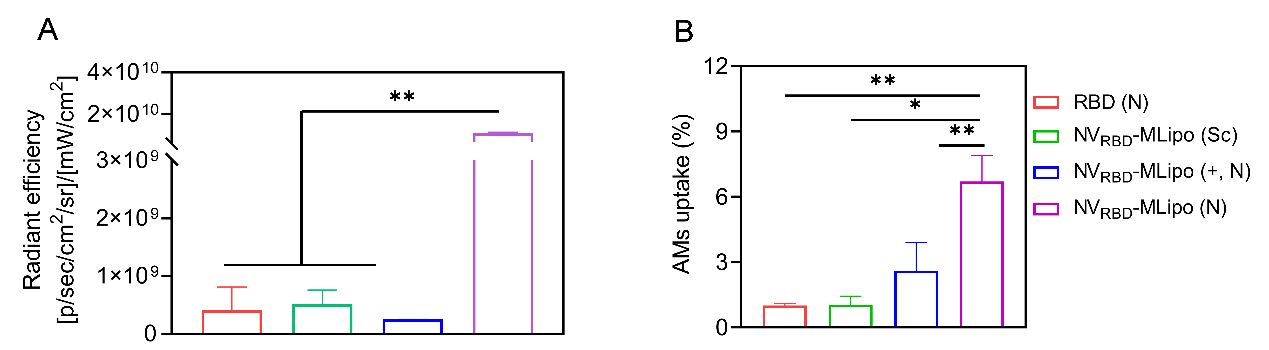


**Fig. S5.** (A) Fluorescence intensity of lung. (B) FCM analysis of various groups uptake in AMs (n = 3). Mice were immunized with different groups as described in Fig. 2D, and lungs were harvested at 12 h (n = 3). Data expressed as means ± SD. *p < 0.05; **p < 0.01.


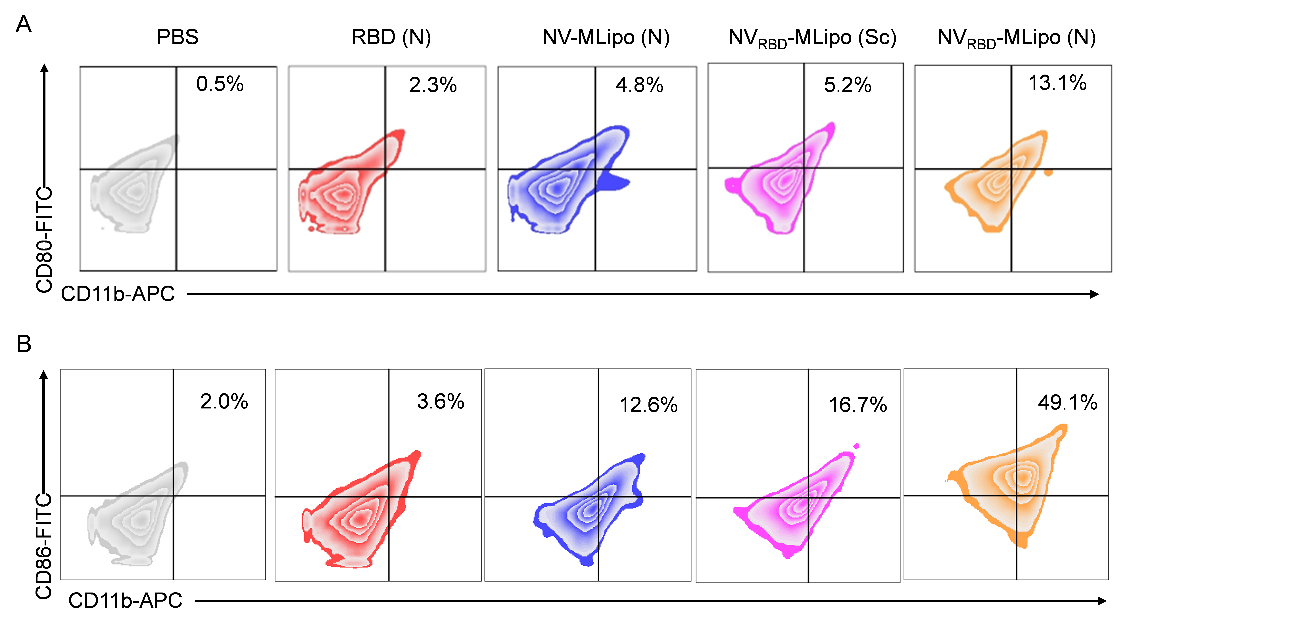


**Fig. S6.** Representative FCM analysis images of CD80^+^ CD11b^+^ (A) and CD86^+^ CD11b^+^ (B) AMs in the lung. Mice were immunized with different groups as described in Fig. 4A, and the lung was collected on day 18 (n = 5).


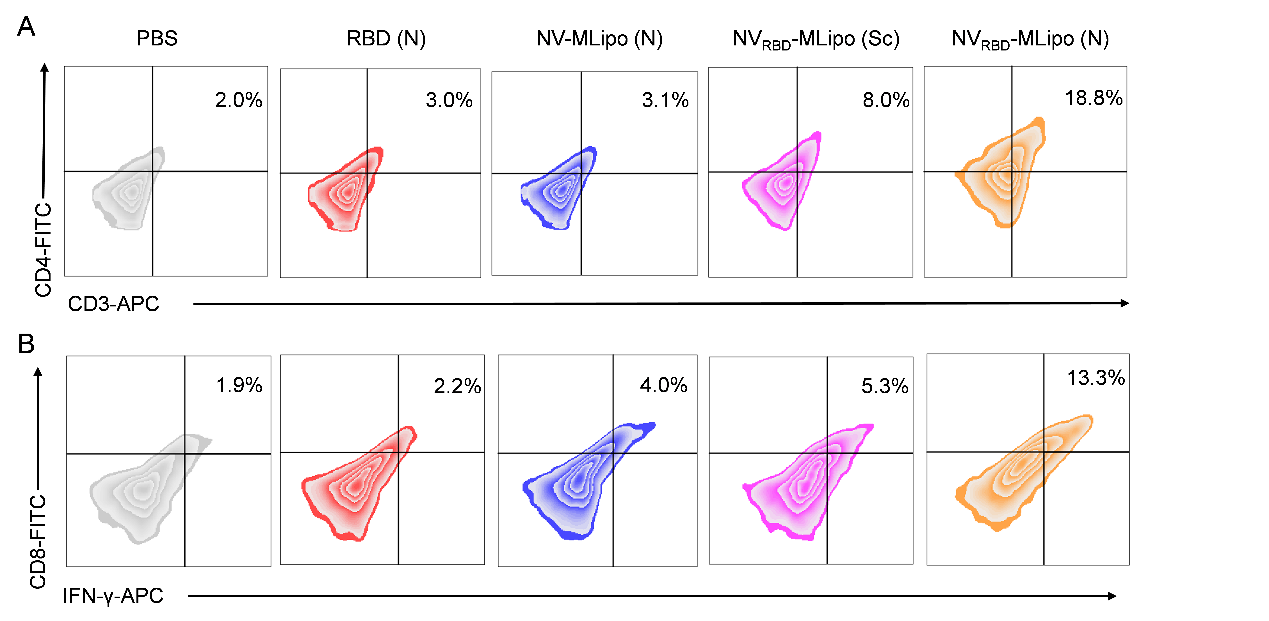


**Fig. S7.** Representative FCM analysis images of CD3^+^ CD4^+^ (A) and IFN-γ^+^ CD8^+^ T cells (B) in the lung. Mice were immunized with different groups as described in Fig. 4A, and the lung was collected on day 18 (n = 5).


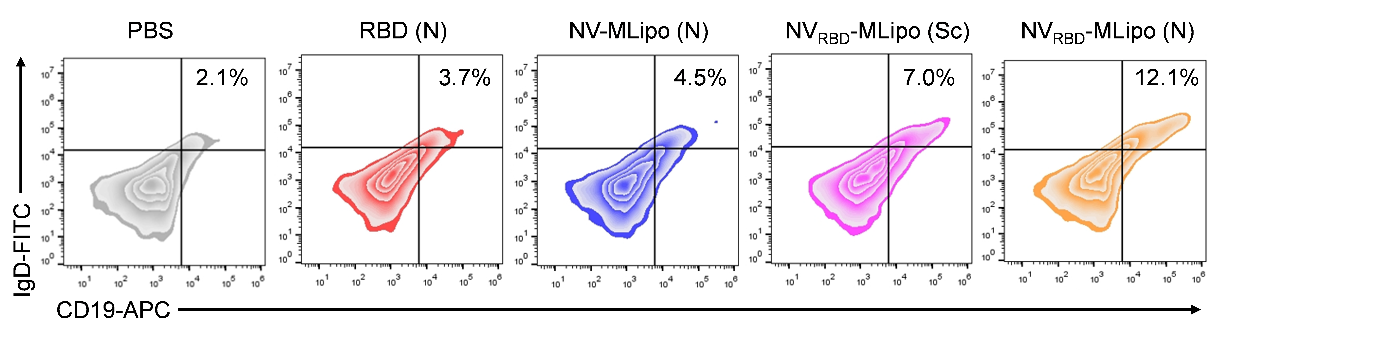


**Fig. S8.** Representative FCM analysis images of CD19^+^ IgD^+^ B cells in the lung. Mice were immunized with different groups as described in Fig. 4A, and the lung was collected on day 18 (n = 5).


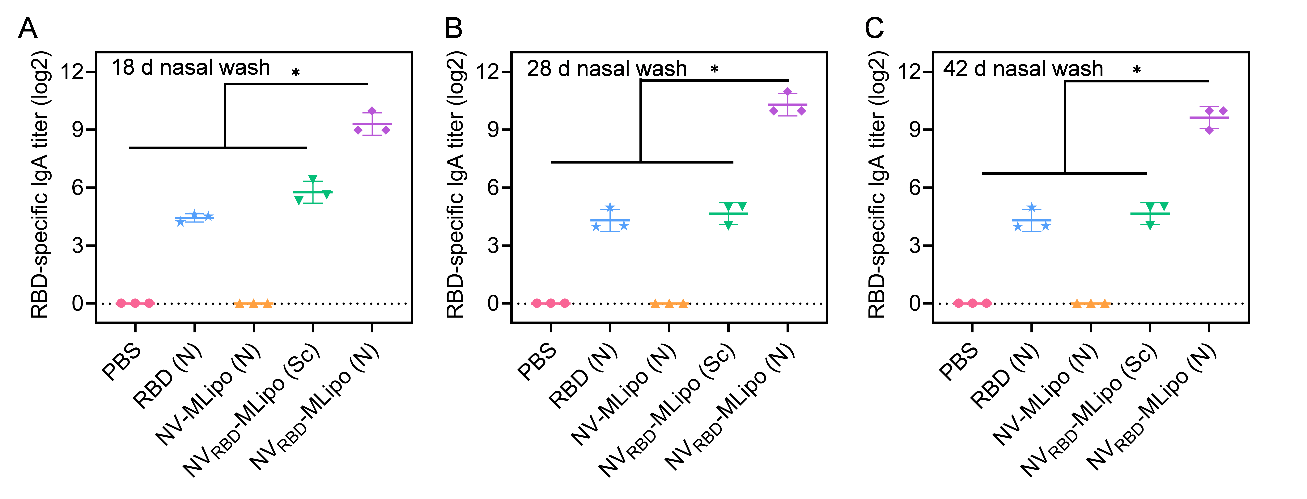


**Fig. S9.** RBD-specific IgA titers in nasal wash assessed by ELISA on days 18 (A), 28 (B) and 42 (C) (n = 4). Data expressed as means ± SD. *p < 0.05.


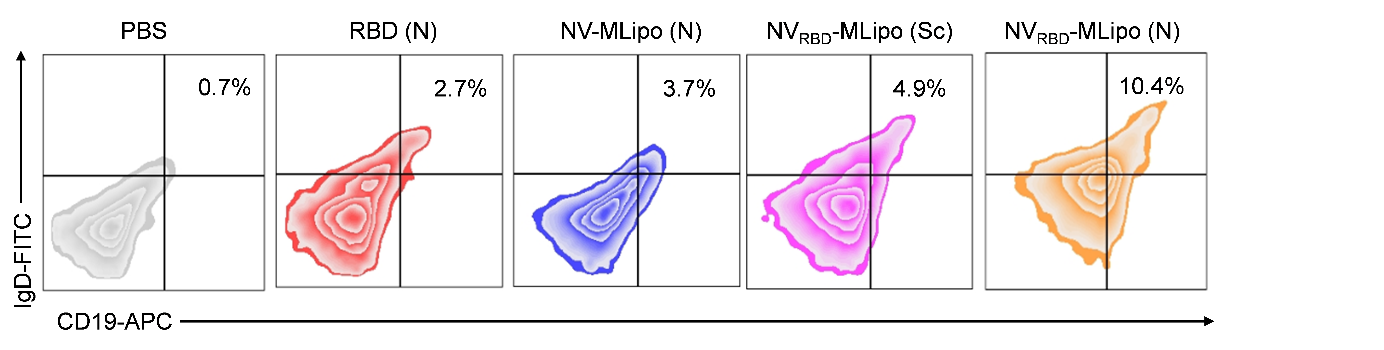


**Fig. S10.** Representative FCM analysis images of CD19^+^ IgD^+^ B cells in spleen. Mice were immunized with different groups as described in Fig. 4A, and spleen was collected on day 18 (n = 5).


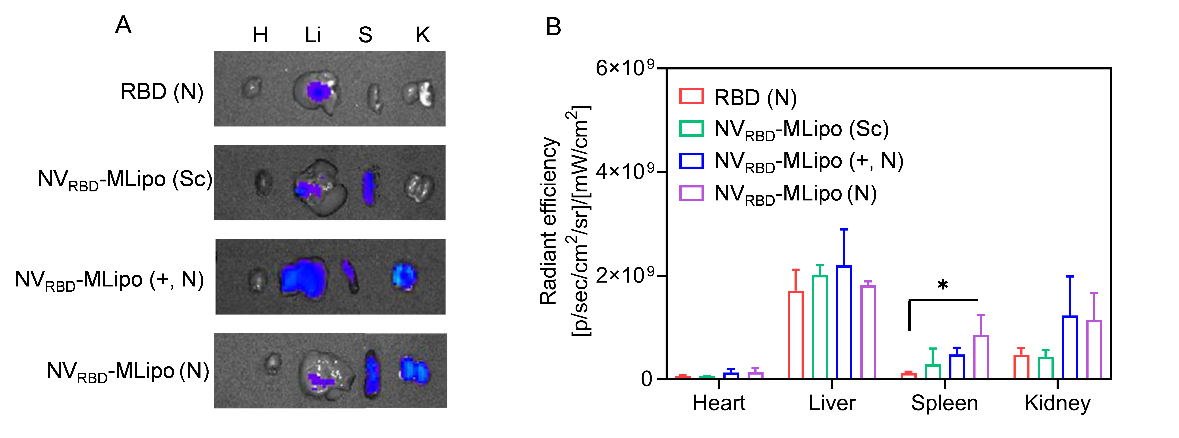


**Fig. S11.** *Ex vivo* fluorescence images (A) and fluorescence intensity (B) of major tissues. Mice were immunized with different groups as described in Fig. 2D, and tissues were harvested at 12 h (n = 3). (H: heart, Li: liver, S: spleen, Lu: lung, K: kidney) Data expressed as means ± SD. *p < 0.05.


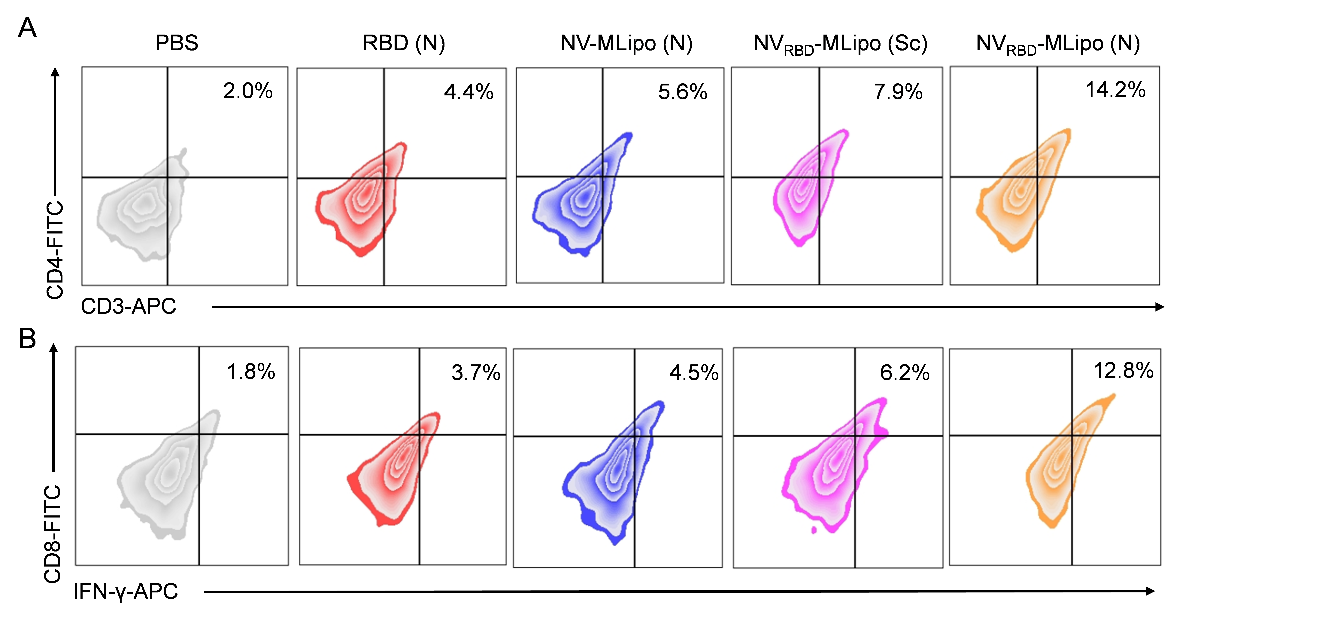


**Fig. S12.** Representative FCM analysis images of CD3^+^ CD4^+^ (A) and IFN-γ^+^ CD8^+^ T cells (B) in spleen. Mice were immunized with different groups as described in Fig. 4A, and spleen was collected on day 18 (n = 5).


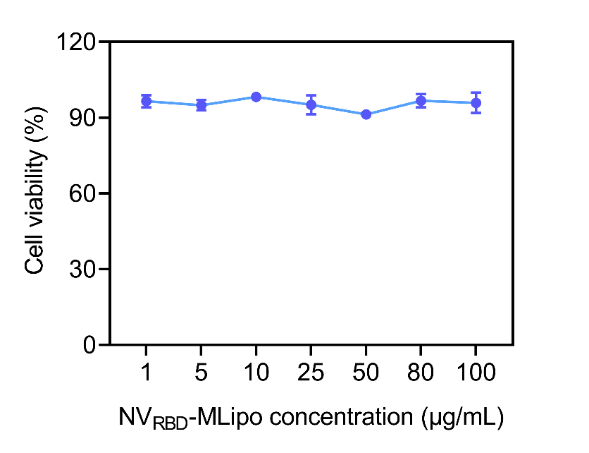


**Fig. S13.** Biosafety of NV_RBD_-MLipo in RAW264.7 cells (n = 3).


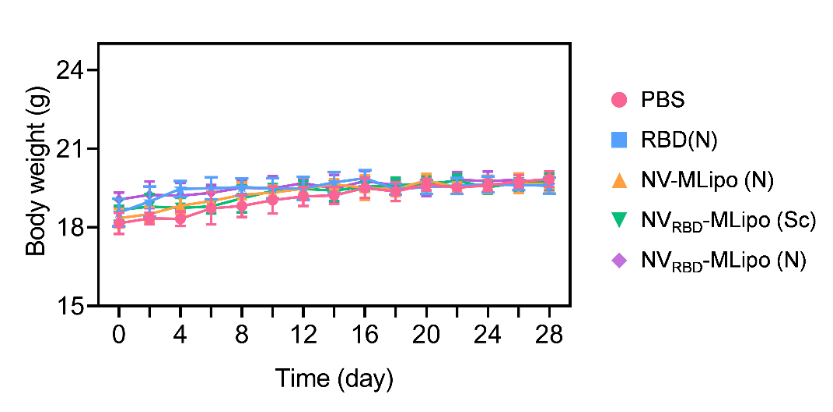


**Fig. S14.** Body weight changes of immunized mice treated as described in Fig. 4A (n = 8).


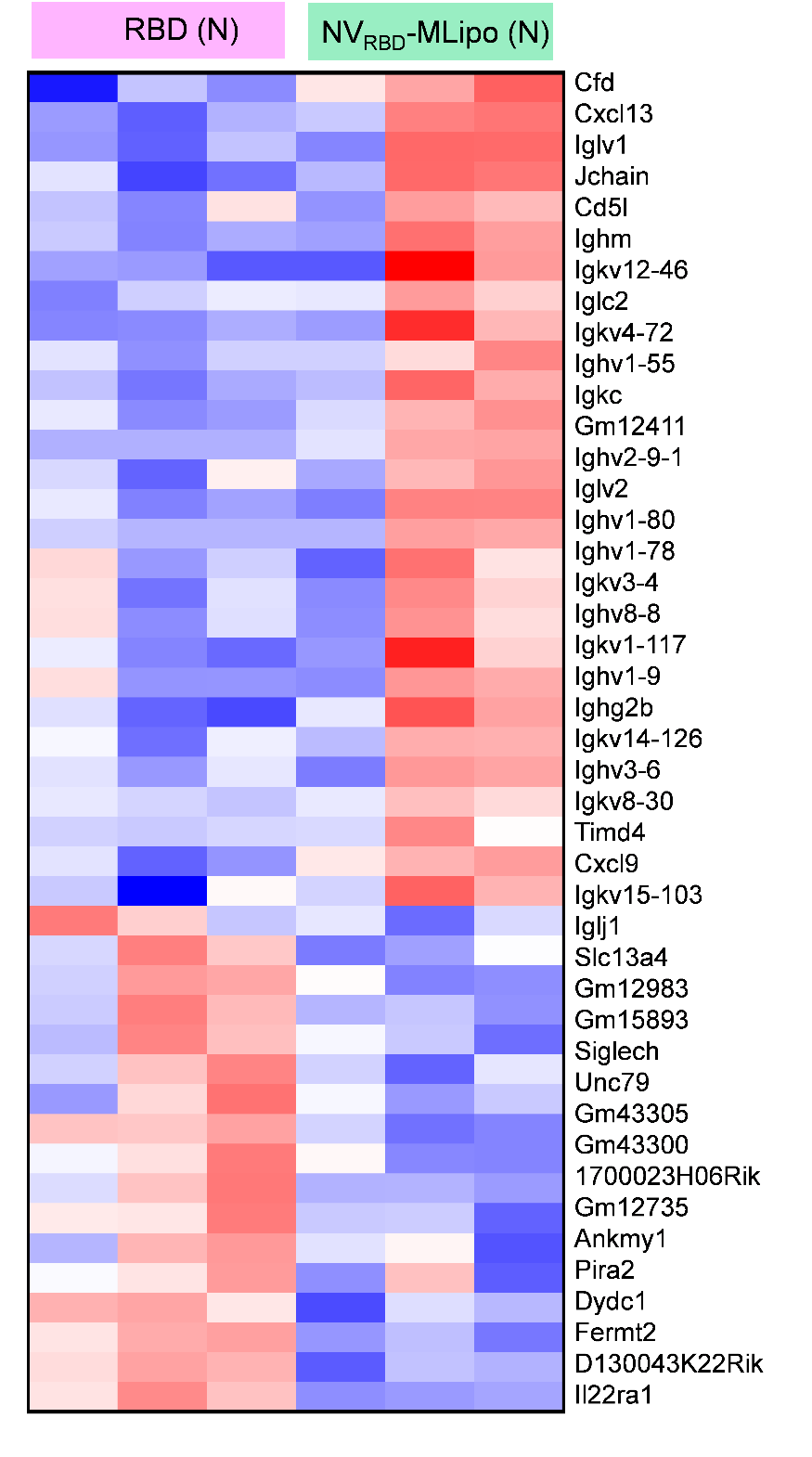


**Fig. S15.** Heat map of differentially expressed genes in lung treated as described in Fig. 8D.
